# Loratadine Combats Methicillin-Resistant *Staphylococcus aureus* by Modulating Virulence, Antibiotic Resistance, and Biofilm Genes

**DOI:** 10.1021/acsinfecdis.3c00616

**Published:** 2023-12-28

**Authors:** Brianna L. Viering, Halie Balogh, Chloe F. Cox, Owee K. Kirpekar, A. Luke Akers, Victoria A. Federico, Gabriel Z. Valenzano, Robin Stempel, Hannah L. Pickett, Pamela M. Lundin, Meghan S. Blackledge, Heather B. Miller

**Affiliations:** † Department of Chemistry, High Point University, High Point, North Carolina 27268, United States; ‡ Department of Biology, High Point University, High Point, North Carolina 27268, United States

**Keywords:** S. aureus, MRSA, antibiotic adjuvant, Stk1, loratadine

## Abstract

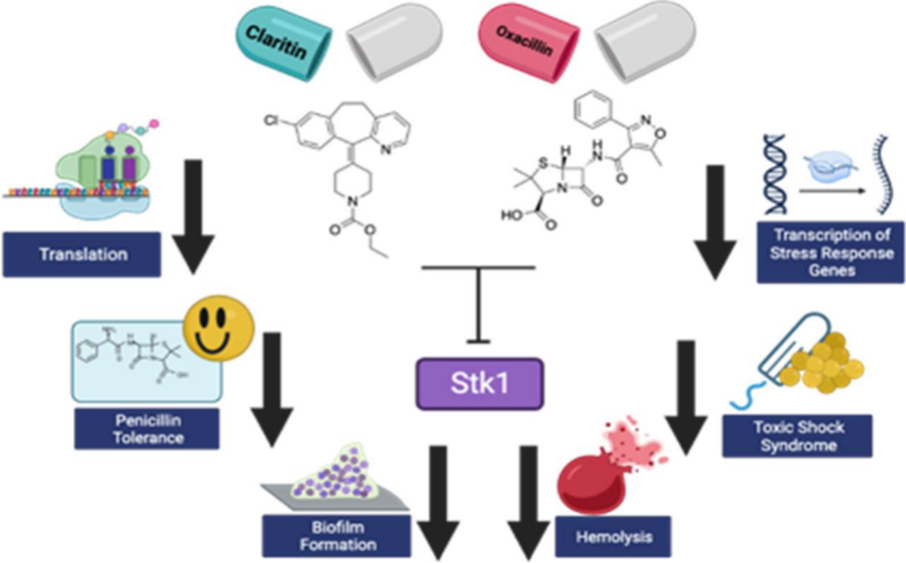

Methicillin-resistant *Staphylococcus aureus* (MRSA) has evolved to become resistant to multiple classes of antibiotics. New antibiotics are costly to develop and deploy, and they have a limited effective lifespan. Antibiotic adjuvants are molecules that potentiate existing antibiotics through nontoxic mechanisms. We previously reported that loratadine, the active ingredient in Claritin, potentiates multiple cell-wall active antibiotics *in vitro* and disrupts biofilm formation through a hypothesized inhibition of the master regulatory kinase Stk1. Loratadine and oxacillin combined repressed the expression of key antibiotic resistance genes in the *bla* and *mec* operons. We hypothesized that additional genes involved in antibiotic resistance, biofilm formation, and other cellular pathways would be modulated when looking transcriptome-wide. To test this, we used RNA-seq to quantify transcript levels and found significant effects in gene expression, including genes controlling virulence, antibiotic resistance, metabolism, transcription (core RNA polymerase subunits and sigma factors), and translation (a plethora of genes encoding ribosomal proteins and elongation factor Tu). We further demonstrated the impacts of these transcriptional effects by investigating loratadine treatment on intracellular ATP levels, persister formation, and biofilm formation and morphology. Loratadine minimized biofilm formation *in vitro* and enhanced the survival of infected *Caenorhabditis elegans*. These pleiotropic effects and their demonstrated outcomes on MRSA virulence and survival phenotypes position loratadine as an attractive anti-infective against MRSA.

Methicillin-resistant *Staphylococcus aureus* (MRSA) is a major human pathogen that has evaded multiple classes of antibiotics for the last few decades.^1,2^ In July 2022, the CDC announced that years of progress in combating antibiotic resistant bacteria were essentially reversed. Infection and death rates increased in hospital settings by approximately 15% from 2019 to 2020. MRSA infection and death rates specifically increased by 13% during that time.^3^ To treat this dangerous pathogen, new antibiotics could be developed. However, this effort is rapidly thwarted by constantly evolving bacterial cells, which readily acquire new resistance genes and virulence mechanisms. Furthermore, large pharmaceutical companies have abandoned most antibiotic discovery programs.^4^ One alternative approach to fighting antibiotic resistant microorganisms is the use of antibiotic adjuvants. These molecules are not toxic to bacterial cells but enhance the efficacy of existing antibiotics often by overcoming the bacteria’s resistance mechanisms.^5^

We have previously studied several repurposed FDA-approved drugs as antibiotic adjuvants *in vitro*.^6,7^ One of these drugs, loratadine, is the active ingredient in Claritin. Loratadine potentiated multiple antibiotics against several strains of MRSA and also hindered biofilm formation.^7^ In that work, we hypothesized that loratadine was inhibiting the activity of the eukaryotic-like serine-threonine kinase Stk1. In *S. aureus*, this master regulator is known to influence both antibiotic resistance and biofilm formation.^8−10^ At the molecular level, we demonstrated that repression of genes in the *bla* and *mec* operons occurred when MRSA cultures were cotreated with both loratadine and oxacillin. Following our initial report, Zheng et al. also showed that loratadine can hinder biofilm formation. Furthermore, they demonstrated that loratadine is capable of entering *S. aureus* cells, and its presence helped mice clear pulmonary infection. They hypothesized that loratadine may disrupt a complex that forms between Stk1 and one of its substrates, MgrA.^11^ This protein is also considered a global transcriptional regulator with known roles in biofilm formation and virulence.^12−14^

Although our previous report provided a great deal of molecular detail surrounding the antibiotic adjuvant activity we were observing, it was limited to measuring a small number of genes based on known roles in resistance. We hypothesized that multiple antibiotic resistance genes were being modulated by loratadine, and by looking transcriptome-wide, additional pathways key to antibiotic potentiation and biofilm inhibition would be revealed. Furthermore, if Stk1 activity was being inhibited by loratadine, then *stk1*-interacting genes and gene products would be modulated, and we could further explore the phenotypic consequences of Stk1 inhibition in the context of these modulated genes and pathways.

## Results

### Hundreds of Genes Are Differentially Expressed after Cotreatment with Loratadine and Oxacillin

**Figure 1:**
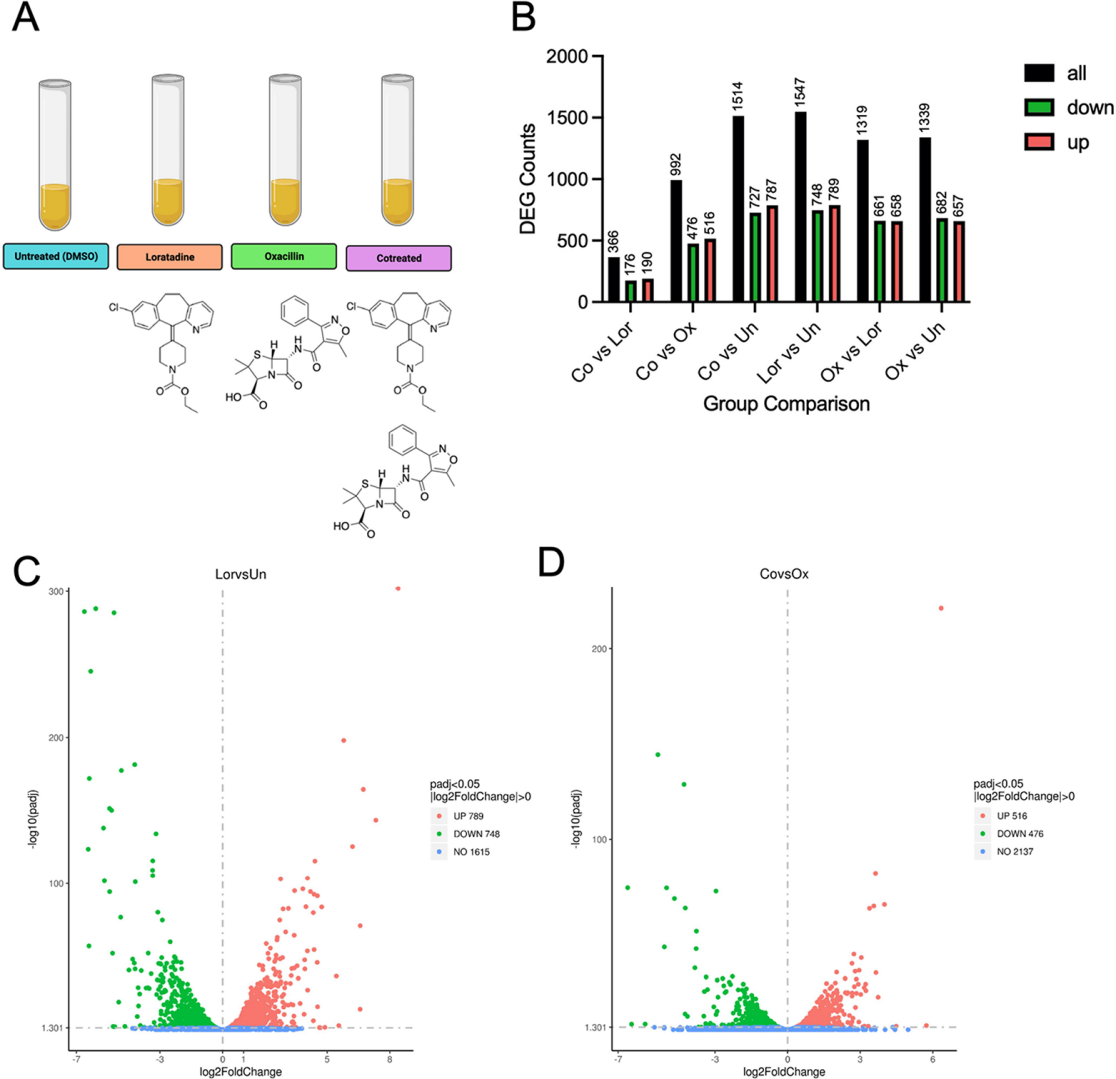
Differential gene expression summary. In all panels, Un represents untreated sample, Lor represents loratadine-treated sample, Ox represents oxacillin-treated sample, and Co represents cotreated sample. (A) MRSA cultures were treated four different ways to examine DEGs. (B) Six different pairwise comparisons could be made. The number of DEGs is shown for each pairwise comparison. (C) A volcano plot illustrates DEGs when comparing loratadine to untreated samples (padj < 0.05) and (D) when comparing cotreated to oxacillin-treated samples (padj < 0.05).

We previously reported loratadine potentiating oxacillin activity in MRSA strain ATCC 43300. Cotreating cells for 1 h with loratadine at 50 μM and oxacillin at 4 μg mL^–1^ reduced the minimum inhibitory concentration (MIC) from 32 to only 1 μg mL^–1^.^7^ Therefore, we treated this strain with the same conditions for RNA-seq experiments. This approach has recently been successful in uncovering transcriptome-wide alterations in gene expression due to antibiotic adjuvant compounds used alone and in combination with antibiotics.^15^ Our experimental design allowed us to measure differentially expressed genes (DEGs) that occur when *S. aureus* cultures were treated with loratadine, oxacillin, or both in combination (Figure 1A).We primarily focused on two pairwise comparisons: (1) loratadine compared to untreated and (2) cotreated compared to oxacillin treated cells. We will refer to these comparisons as Lor vs Un and Co vs Ox. The Lor vs Un comparison reveals DEGs due to loratadine treatment alone. Co vs Ox identifies DEGs uniquely affected by cotreatment with loratadine and oxacillin rather than those affected by oxacillin alone. Antibiotic treatment is known to alter gene expression in MRSA as the bacteria attempts to survive. Comparing cotreatment of oxacillin and loratadine to treatment with oxacillin alone allows us to interrogate how loratadine may impact MRSA’s ability to respond to antibiotic treatment through modulating mRNA levels.

As shown in Figure 1B, hundreds of DEGs are revealed in each of the six pairwise comparisons. When focusing just on loratadine treatment, we found that 789 genes were significantly upregulated, whereas 748 genes were significantly downregulated (Figure 1C). This represents approximately 49% of measured mRNAs being affected by 50 μM loratadine treatment. Cotreatment showed that 516 genes were significantly upregulated, whereas 476 genes were significantly downregulated (Figure 1D). This equates to approximately 32% of measured mRNAs affected by loratadine and oxacillin used in combination that were not affected by oxacillin alone.

### Subcluster Analysis Showed Different Patterns of Gene Expression Changes across Treatments

**Figure 2:**
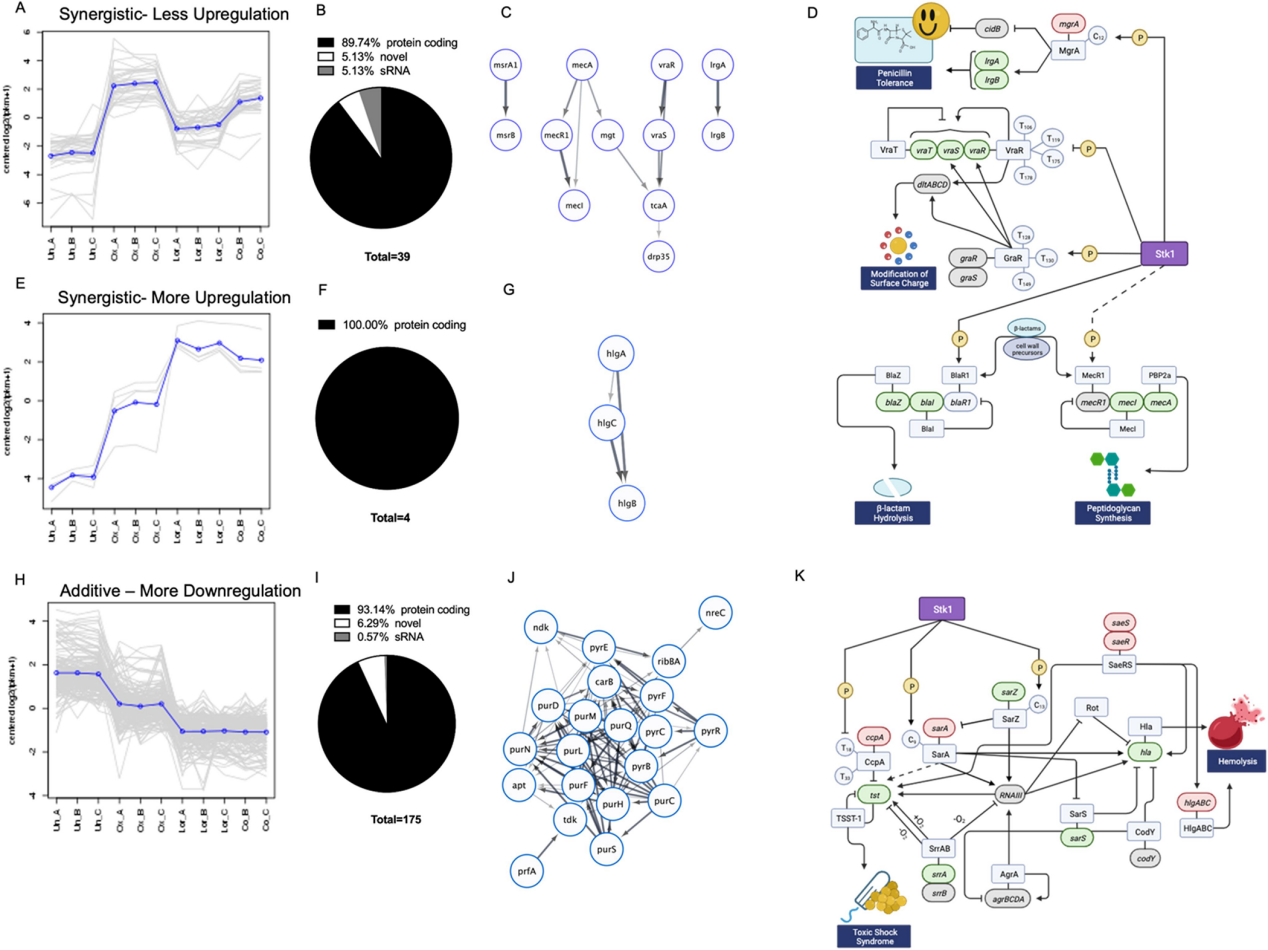
Subclusters of differentially expressed genes. (A) Differentially expressed genes (*n* = 39, padj ≤0.05) that show less upregulation with cotreatment compared to oxacillin alone. In each panel, the *x* axis plots each individual sample, where Un represents untreated sample, Lor represents loratadine-treated sample, Ox represents oxacillin-treated sample, and Co represents-cotreated sample. Each biological replicate is labeled A, B, or C. The *y* axis plots the centered log2 fpkm (fragments per kilobase per million mapped fragments) with the blue line indicating the average of all genes in the subcluster. (B) A breakdown by percentage of protein coding, novel, and small RNAs (sRNAs) in the 39 genes in the subcluster shown in panel A. (C) STRING network illustrating protein interactions among genes in the subcluster shown in panel A. Single nodes were removed. (D) Key genes and proteins known to impact *S. aureus* antibiotic resistance are shown. Transcriptional changes with cotreatment as compared to oxacillin treatment are indicated by gene shading. Genes that are downregulated are colored in green, genes that are upregulated are colored in red, and genes that show no significant change from untreated are in gray. Although genes in the *bla* operon were not detected in our RNA-seq experiment, we have previously reported the effects of loratadine on these genes in ATCC 43300.^7^ (E) Differentially expressed genes (*n* = 4, padj ≤ 0.05) that show more upregulation with cotreatment compared to oxacillin alone. (F) A breakdown by percentage of protein coding genes in the four genes in the subcluster shown in panel E. (G) STRING network illustrating protein interactions among genes in the subcluster shown in panel D. *ydfJ* was a single node and was removed. (H) Differentially expressed genes (*n* = 175, padj ≤0.05) that show more downregulation with cotreatment compared to oxacillin alone. (I) A breakdown by percentage of protein coding, novel, and small RNAs (sRNAs) in the 175 genes in the subcluster shown in panel H. (J) STRING network illustrating protein interactions among genes in the subcluster shown in panel H. Single nodes were removed. (K) Key genes and proteins known to impact *S. aureus* toxins and virulence are shown. Transcriptional changes with loratadine compared to untreated samples are indicated by gene shading. Genes that are downregulated are colored in green, genes that are upregulated are colored in red, and genes that show no significant change from untreated are in gray.

Given our previous work on loratadine-induced antibiotic potentiation,^7^ we hypothesized that DEGs would include key antibiotic resistance genes as well as virulence factors. These hundreds of widespread changes in gene expression were next categorized into subclusters based on the patterns of up- and downregulation measured in each of the treated samples. The first subcluster was populated with 39 genes that dramatically increased in expression with oxacillin challenge (Figure 2A). Loratadine treatment resulted in a less dramatic increase, and cotreatment resulted in expression somewhere in the middle. We call this subcluster’s pattern “Synergistic, Less Upregulation” because cotreatment modulates gene expression to levels that are less than those of cells treated with oxacillin alone. The genes in this subcluster were primarily protein-coding, but approximately 5% were likely novel protein-coding RNAs, and another 5% were likely small RNAs (sRNAs) (Figure 2B). Importantly, many of the genes that were modulated in this fashion were major antibiotic resistance genes including *mecA*, *mecI*, and *mecR1,* as illustrated by the interaction network created from this subcluster (Figure 2C). This is consistent with previous RT-qPCR results that we reported demonstrating that the *mec* operon is repressed in loratadine-induced antibiotic potentiation.^7^ However, there were other genes affected as well. These include the two-component system (TCS) *vraR/S*, which plays a major role in the *S. aureus* response to cell-wall active antibiotics and is an Stk1 substrate.^16^ Two other genes, *lrgA* and *lrgB,* encode holin-like proteins that are regulators of cell death and promote penicillin tolerance.^17^ Recently, LrgA has also been shown to help transport carbohydrate metabolism byproducts.^18^ Finally, *msrA1* and *msrB* encode cotranscribed peptide methionine sulfoxide reductases. These enzymes serve to minimize damage to proteins during oxidative stress. Both have previously been shown to be elevated after oxacillin treatment.^19^ Our data also show upregulation of *msrA1 and msrB* after oxacillin treatment, and both genes are downregulated when cotreated with loratadine. Although the STRING database from which the interaction networks were derived does not display a known interaction between *msrA1/B* and *vraR/S*, others have reported that the VraR/S TCS helps regulate these methionine sulfoxide reductase responses to cell wall-active antibiotics.^20^ The functional enrichment of this interaction network was highly significant (*p* = 1.55 × 10^–9^), indicating that more interactions among gene products are found here than would be due to chance alone with a similarly sized group of gene products in the *S. aureus* genome. Figure 2D summarizes these results and illustrates key interactions between regulatory genes and proteins, lending additional support to our hypothesis that loratadine inhibits Stk1 leading to the observed downstream gene expression changes.

The second subcluster contained only four DEGs. These all increased in expression with oxacillin, increased even further with loratadine, and decreased only slightly with cotreatment compared to loratadine. We call this pattern “Synergistic, More Upregulation” (Figure 2E). All four DEGs were protein-coding (Figure 2F), and three were found to encode gamma hemolysin components (Figure 2G). Gamma hemolysin components A, B, and C are located in two different operons and function as exotoxins to lyse host red blood cells.^21^ As expected,^22,23^ their expression increased because of antibiotic presence, but loratadine then elevated expression levels even higher. The fourth gene in this subcluster, *ydfJ*, encodes a putative transmembrane protein pump but was excluded from the interaction network because it was not connected physically or functionally to the other three gene products. The differential expression of *ydfJ* was higher in both magnitude and statistical significance than any other mRNA (Co vs Ox revealed a log2 fold change of 6.34, padj = 6.42 × 10^–222^). This interaction network’s functional enrichment was also found to be highly significant (*p* = 4.72 × 10^–7^).

The final subcluster contained 175 DEGs. These decreased in expression with oxacillin treatment, decreased further with loratadine alone, and decreased even further when both drugs were used in combination (Figure 2H). We refer to this pattern as “Additive, More Downregulation”. As expected, most of these genes encoded proteins, but several novel genes and sRNAs were also detected via RNA-seq (Figure 2I). The gene for toxic shock syndrome toxin-1 (*tst*) was one of the most downregulated genes with cotreatment (log2 fold change = −5.00, padj = 4.61 × 10^–75^) and was categorized to this subcluster. Although not all DEGs are represented in the interaction networks (a limitation that is true for all studies due to less than complete representation in the STRING database), the network formed in Figure 2J reveals a strong tie to purine and pyrimidine biosynthesis genes (*p* = 1.00 × 10^–16^). Figure 2K illustrates that loratadine alone modulates genes in addition to those involved in antibiotic resistance, including the major virulence factor *tst,* and many genes involved in nucleotide metabolism and hemolysis. Furthermore, the subcluster analysis illustrates that there are multiple “trends” of DGE occurring simultaneously in the cell, all of which are elicited by loratadine and could be at least partially explained by Stk1 inhibition.

### Gene Expression Changes Detected with RNA-seq Were Independently Validated with RT-qPCR

**Figure 3:**
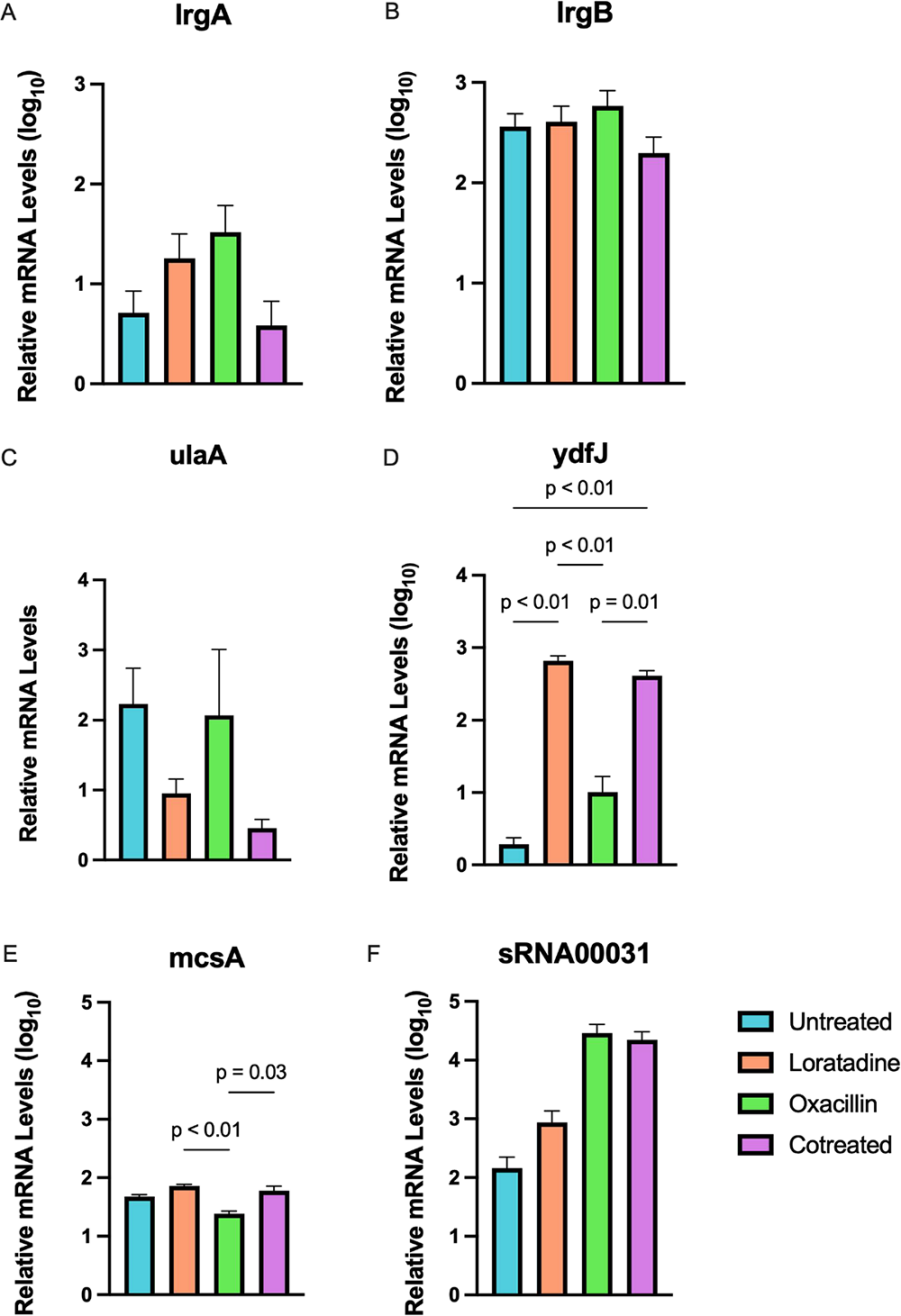
Differentially expressed genes were validated by RT-qPCR. (A) *lrgA*, (B) *lrgB*, (C) *ulaA*, (D) *ydfJ*, (E) *mcsA*, and (F) *sRNA00031*. In all panels, the *y* axis displays the average mRNA levels relative to *16S* rRNA. Error bars represent the standard error of the mean. Adjusted *p* values ≤ 0.05 are shown.

The high-throughput nature of RNA-seq experiments is extremely powerful in identifying gene expression changes transcriptome-wide. However, they inevitably contain both false positives and false negatives. To help validate the effects that were measured, we used RT-qPCR as a complementary technique to measure and confirm a subset of DEGs. These experiments were performed on MRSA 43300 cultures treated and purified independently from those used in RNA-seq Furthermore, the subset included both up- and downregulated genes observed with cotreatment. For consistency, loratadine was used at 50 μM, and oxacillin was used at 4 μg mL^–1^. Incubation was performed at 37 °C with shaking for 1 h. As shown in Figure 3A,B, both *lrgA* and *lrgB* mRNA levels increased with oxacillin and loratadine treatment separately but decreased when the two drugs were used in combination. This was consistent with the trend in Co vs Ox found in the RNA-seq data. *ulaA*, the ascorbate-specific transferase component of the phosphotransferase system; *ydfJ*, a putative membrane transport protein; and *mcsA*, a gene that encodes a protein arginine kinase activator protein, all matched the Co vs Ox modulation that was found in RNA-seq data (Figure 3C–E). We also validated a small RNA (sRNA00031) that showed loratadine-induced modulation. As shown in Figure 3F, this sRNA is dramatically upregulated with oxacillin treatment but less so with loratadine. Whereas sRNAs are underannotated in *S. aureus* genomes, our RNA-seq approach revealed three additional putative sRNAs that were differentially expressed. All MRSA strains in the staphylococcal regulatory RNAs, BSRD, and Rfam databases were examined for these four sRNA sequences, but none were found. This suggests that they are novel sRNAs. IntaRNA analysis^24^ predicted that all four sRNAs target the same hypothetical protein with little information available concerning its function. However, Uniprot’s peptide search revealed a highly conserved protein in *S. aureus* similar to the yeast Mid2-like cell wall stress sensor domain protein (data not shown). Functional details about this protein or sRNAs that regulate it are absent in the literature. Together, these experiments resulted in a 100% validation rate. This supports the biological changes at the RNA level, giving further confidence in additional conclusions drawn from this high-throughput data.

### Loratadine Treatment May Subtly Change *stk1* and *stp1* mRNA Levels

Among the hundreds of modulated genes, we investigated changes in *stk1* itself as well as its cognate phosphatase serine-threonine phosphatase 1 (*stp1*). The mRNA levels of *stk1* showed a subtle decrease when loratadine was administered either alone or in combination with oxacillin. These statistically significant decreases were always smaller in magnitude than −1.0 log2 fold change, which likely explain why only decreases with cotreated cells were captured when attempted with RT-qPCR here (Figure S3). RNA-seq showed that *stp1* expression also decreased with loratadine compared to untreated cells (log2 fold change = −1.27, padj = 1.66 × 10^–8^) (Table S4) and with cotreatment compared to oxacillin (log2 fold change = −1.04, padj = 7.79 × 10^–6^) (Table S5). These changes were recapitulated with RT-qPCR (Figure S3). Together, these suggest that loratadine may subtly lower *stp1* and *stk1* mRNA levels by an unknown mechanism in this strain.

### MRSA Cultures Cotreated with Loratadine and Oxacillin Have Disrupted Metabolic Pathways and Ribosomal Gene Expression

Given the large number of DEGs upon loratadine treatment, we next analyzed the data for commonalities that would begin to provide biological context. Focusing on just upregulated genes in the loratadine-treated compared to untreated samples revealed two Gene Ontology (GO) categories that were significantly enriched. These were the biological process of oxidation–reduction (GO: 0055114, *n* = 68, padj = 0.034) and the molecular function of oxidoreductase activity (GO: 0016491, *n* = 72, padj = 0.035) (Table S11). Among the downregulated genes, the biological processes of transport (GO: 0006810, *n* = 90, padj = 0.012) and translation (GO: 0006412, *n* = 32, padj = 0.012) were statistically enriched (Table S12). This supports loratadine treatment alone modulating redox, cellular transport, and translation.

When we analyzed the upregulated genes that were detected upon cotreatment compared to oxacillin alone, significant enrichment was found in the cellular compartment categories of intracellular nonmembrane-bounded organelle (GO: 0043232, *n* = 15 genes, padj = 0.017) and ribonucleoprotein complex (GO: 1990904, *n* = 13 genes, padj = 0.024) (Table S13). Although these two cellular compartments contain a high degree of overlap, the ribonucleoprotein complex category genes are not fully contained within the first category. This enrichment again points to ribosomal genes being modulated by loratadine and oxacillin cotreatment more than would be expected due to chance alone. There were no enriched GO categories among downregulated genes in cotreated compared to oxacillin-treated samples (Table S14).

Another bioinformatic tool used to look for commonalities in transcriptomic experiments is enrichment of genes belonging to Kyoto Encyclopedia of Genes and Genomes (KEGG) pathways. Analyzing all DEGs (regardless of up- or downregulation) in loratadine vs untreated samples revealed no statistically significant enrichment (Table S15). However, parsing out only genes upregulated by loratadine showed many mapping to panthothenate and CoA biosynthesis (sau00770, *n* = 14, padj = 0.0013), carboxylate and dicarboxylate metabolism (sau00630, *n* = 14, padj = 0.0089), starch and sucrose metabolism (sau00500, *n* = 11, padj = 0.0089), and microbial metabolism in diverse environments (sau01120, *n* = 55, padj = 0.010, Table S16). Genes downregulated by loratadine mapped to the ribosomal pathway (sau03010, *n* = 42, padj = 2.93 × 10^–8^). In addition, purine and pyrimidine metabolism was depressed (sau00230, *n* = 25, padj = 0.023 and sau00240, *n* = 17, padj = 0.023, respectively, Table S17).

**Figure 4:**
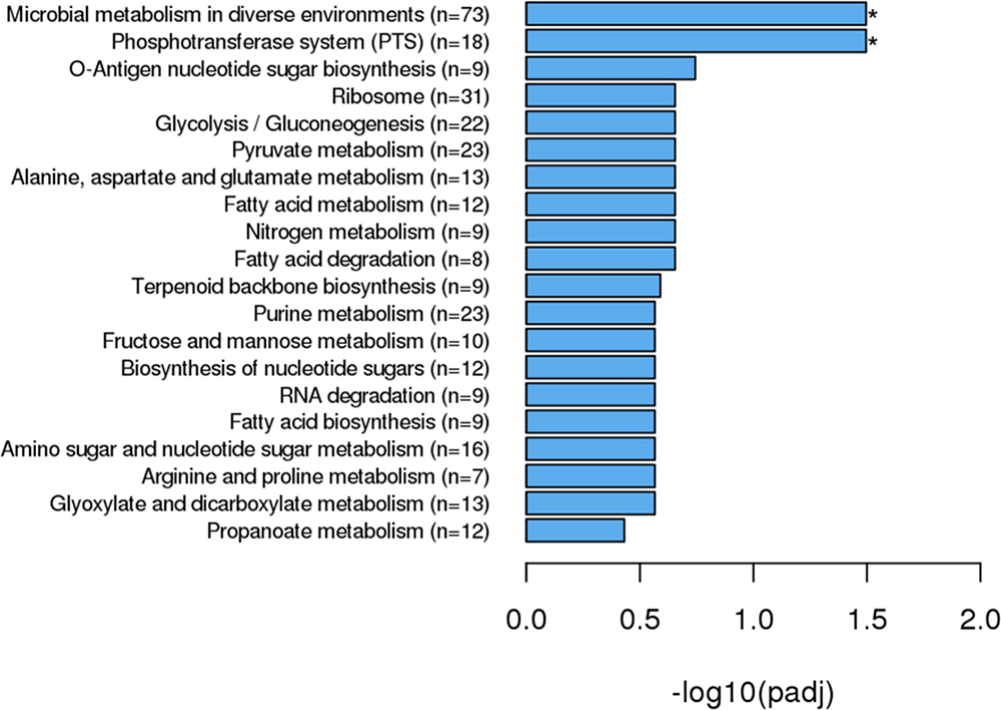
KEGG pathway analysis of all DEGs in cotreated vs oxacillin-treated samples. All genes, regardless of up- or downregulation, were included. *padj ≤0.05.

Analyzing all DEGs (regardless of up- or downregulation) in cotreated compared to oxacillin-treated samples revealed that two KEGG pathways were significantly enriched (Figure 4 and Table S18). Microbial metabolism in diverse environments was the most highly populated (*n* = 73) as well as the most statistically significant (padj = 0.032). The phosphotransferase system (PTS) was the second most significantly enriched pathway (*n* = 18, padj = 0.032). The DEGs that mapped to the PTS play a role in transport of at least eight different mono- and disaccharides as well as phosphoenolpyruvate (PEP) in *S. aureus* cells (Table S18 and Figure S4). Consistent with GO results, the KEGG analysis also revealed a tie to ribosomal genes, with the ribosome being one of the most populated pathways (*n* = 31, padj= 0.22) (Figure 4 and Table S18).

When parsing out upregulated genes only, we again observed enrichment in the ribosomal pathway (sau03010, *n* = 28, padj = 3.06 × 10^–6^, Table S19 and Figure S5). Among only downregulated genes, cotreatment resulted in a significant enrichment in genes involved in the terpenoid backbone biosynthesis pathway (sau00900, *n* = 9, padj = 0.032, Table S20 and Figure S6). Terpenoids are essential for the formation of bacterial cell walls, suggesting that downregulating their expression is one of the ways that loratadine counters the effects of cell-wall active antibiotics like oxacillin.

**Figure 5:**
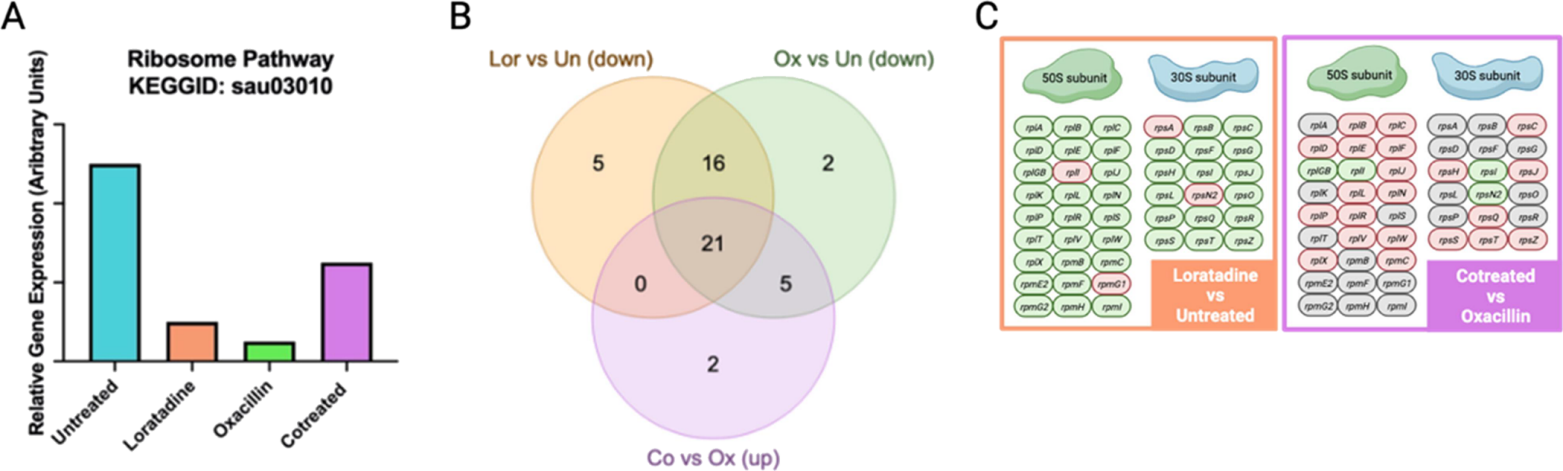
Trend in ribosomal pathway gene regulation. (A) Each experimental treatment compared to the untreated control revealed a statistically significant enrichment (padj ≤0.05) in DEGs mapping to the KEGG ribosome pathway. Arbitrary units were assigned to illustrate this trend as a bar chart across all four treatments rather than just in pairwise comparisons. (B) Numbers represent ribosomal DEGs unique or common between pairwise comparisons. Un represents untreated samples, Lor represents loratadine-treated samples, Ox represents oxacillin-treated samples, and Co represents cotreated samples. (C) Ribosomal DEGs are illustrated according to ribosomal subunit. Red indicates upregulation and green indicates downregulation in the pairwise sample comparison listed.

Persisters are a subpopulation of metabolically dormant bacteria^25^ that are unique from antibiotic-resistant or -tolerant cells.^26^ The emergence and maintenance of persister cells have been shown to be regulated at the translational level. Translation is lower in persisters compared to actively growing cells.^27,28^ Because ribosomal genes were heavily affected in multiple treatment comparisons, we examined this modulation across all drug treatments to begin investigating loratadine’s potential role in persistence. When each treatment was compared to the untreated control, a clearer trend in ribosomal gene regulation was revealed. As shown in Figure 5A, loratadine and oxacillin treatment individually resulted in downregulated genes found in the ribosomal pathway. However, when the two drugs were used in combination (cotreated), expression of these ribosomal genes increased toward normal, untreated levels. Figure 5B,C shows that the majority of these DEGs, 21, that were downregulated with either drug were the same DEGs upregulated in the cotreated samples. Given this trend, it is possible that cotreatment of oxacillin with loratadine as an adjuvant counteracts the bacterial cells’ attempt to reach persistence.

### Interaction Network Analysis Supports Stk1 Being a Molecular Target of Loratadine

These bioinformatic analyses add critical functional context to large lists of DEGs. However, pathways operate in complex networks and not in isolated events devoid of cross talk. Therefore, interaction networks were next used to better visualize interconnections and identify those gene products that were more densely connected than others. This tactic was also used to help support or refute our hypothesis that loratadine is targeting and inhibiting the master regulator Stk1 given that many Stk1 targets are known.

**Figure 6:**
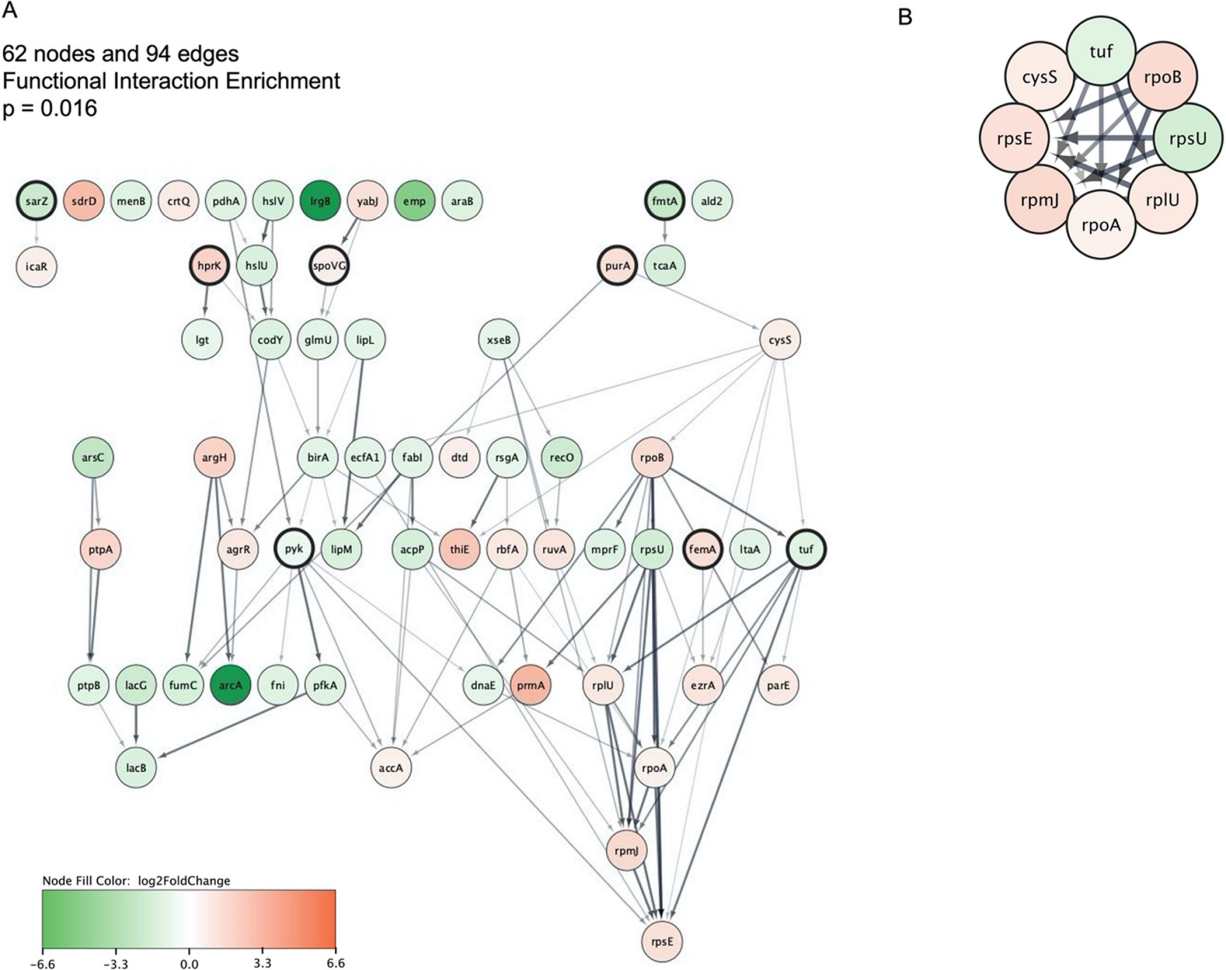
Interaction network of gene products modulated by loratadine in the presence of oxacillin. (A) Nodes with darker outlines are known Stk1 interactors. (B) The top-scoring MCODE cluster is shown. In both panels, node colors are based on log2 fold change. Edges were styled to reflect STRING database scores, where thicker lines indicate larger scores. An interactive version of this network that can be viewed within Cytoscape is found on NDex at https://www.ndexbio.org/#/network/fee90103-410c-11ee-aa50-005056ae23aa.

To extract as many meaningful interactions from our transcriptomic analysis as possible, we took advantage of the four unique treatments and created two separate interaction networks. The first consisted of DEGs in the STRING database that were significantly affected in cotreated samples compared to oxacillin-treated samples. This network consisted of 308 nodes (gene products) and 2934 edges (either functional or physical interactions known to exist). The functional interaction enrichment was highly statistically significant (*p* = 1 × 10^–16^), indicating that this group of genes was more interconnected than would be expected due to random chance (Figure S7). The second interaction network consisted of DEGs that were significantly affected in loratadine-treated samples compared to untreated samples. This network had 443 nodes and 6126 edges. The functional interaction enrichment was also highly statistically significant (*p* = 1 × 10^–16^) (Figure S8). As a downregulated gene, *stk1* can be found in each of these two networks. Stk1’s central location in these layouts (yellow nodes) and its first neighbors (nodes that connect directly with *stk1* via an edge) are highlighted. There were 50 and 52 edges in the first and second networks, respectively, connecting *stk1* to known interactors that were also differentially expressed. For context, the average number of edges from a node in these networks was only 19 and 28, respectively. These results support the hypothesis that as loratadine decreases *stk1* and *stp1* mRNA levels (and potentially protein activity), it triggers widespread gene expression changes throughout the cell. Lastly, we merged these two existing networks to reveal DEGs that occurred with cotreatment but not with loratadine alone. This stringent filtering step served to reveal DEGs that were uniquely modulated with loratadine in the presence of oxacillin, which is the clinically relevant scenario for a molecule serving as an antibiotic adjuvant. As shown in Figure 6A, 62 nodes and 94 edges remained. The functional interaction enrichment was statistically significant (*p* = 0.016).

Strikingly, at least eight known *stk1* interactors were still found in this filtered network (darker outlined nodes): *sarZ*,^29^*fmtA*,^30^*hprK*,^31^*spoVG*,^32^*purA*,^33^*pyk*,^34^*femA*,^35^ and *tuf*.^31^ The mRNA levels of *sarZ* were downregulated. This protein is a transcriptional regulator of virulence genes, including alpha and beta hemolysins and RNAIII (delta hemolysin).^36^ It also interacts with *icaR*, a gene whose protein represses biofilm formation.^37^ The mRNA levels of *icaR* were shown to be upregulated by cotreatment. Additionally, several genes in this merged interaction network have roles in antibiotic resistance: *fmtA*,^38^*tcaA*,^39^*mprF*,^40,41^ and *femA*,^42^ as revealed by Uniprot keyword enrichment (false discovery rate = 0.035). Consistent with the GO and KEGG enrichment results reported here, many modulated ribosomal genes also appear in the network.

Dense networks offer a challenge in identifying those nodes (DEGs) that are most influential in downstream effects of an experimental treatment. To help identify and prioritize nodes as potential regulators in the signaling events triggered uniquely by loratadine and oxacillin, we used tools within the Cytoscape software to identify network hubs. These hubs have the most edges (interactions) within the network. Both cytoHubba and MCODE gave identical results in reporting hub genes. Figure 6B shows the top scoring cluster, containing eight nodes. Most of these genes were upregulated, with the exceptions of elongation factor Tu (*tuf*) and 50S ribosomal protein L21 (*rplU*). The list of hub genes was extended to rank the top 10 (Table S24). The two top-ranking genes affected uniquely by cotreatment were both subunits of DNA-directed RNA Polymerase. This finding represents yet another major transcriptional effect that cotreatment causes in these cells. In terms of translational effects, the ribosomal protein genes were found in both the small and large ribosomal subunit. An additional tie to translation was the cysteine–tRNA ligase (*cysS*). The glycolytic enzyme pyruvate kinase (*pyk*) and the bifunctional ligase/repressor (*birA*) also make the list of top 10 hub genes in this network. Together, this supports the pleiotropic effects of loratadine in changing MRSA’s transcriptional as well as translational repertoire during antibiotic challenge. Whereas only *tuf* and *pyk* are previously reported interactors of *stk1*, this list of hubs may contain genes that have great influence downstream of Stk1 inhibition. These hubs will remain a focus of future studies to further elucidate the molecular consequences of loratadine-treated MRSA cells.

### Loratadine Does Not Enhance Persisters in MRSA 43300

Our high-throughput data revealed enriched categories, pathways, and networks to which DEGs belonged, leading us often to the ribosome. Although suppressing translation, the most energetically expensive cellular process, occurs during antibiotic persistence in *S. aureus*,^27,28^ the full suite of molecular mechanisms involved in persister formation is still being investigated. Accordingly, no categories or pathways exist that contain genes involved in persister formation. Therefore, we mined the literature to look for genes attributed to *S. aureus* antibiotic persisters to determine if any DEGs affected by loratadine were present. Strikingly, 15/19 genes we identified as related to persisters showed significant modulation with loratadine or loratadine in combination with oxacillin, with 13/15 being downregulated. Cotreatment with loratadine and oxacillin often resulted in a more dramatic downregulation of mRNA levels than loratadine alone (Table S25). These DEGs can be visualized in Figure S9.

**Figure 7:**
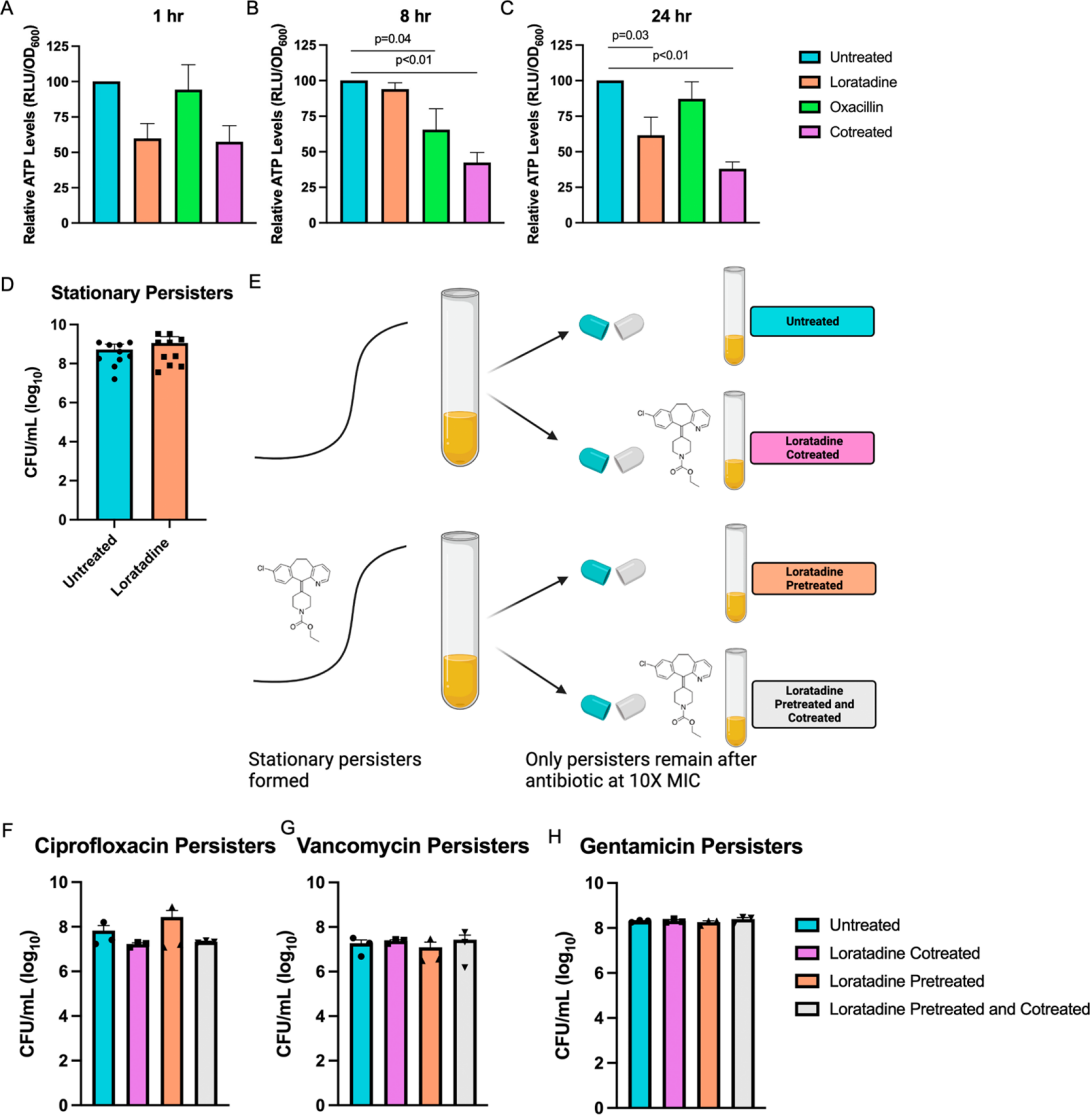
Loratadine reduces ATP but not persisters in MRSA 43300. Intracellular ATP levels are shown relative to the untreated control at (A) 1 h, (B) 8 h, or (C) 24 h of treatment. In panels A–C, average values from four independent experiments are shown, with error bars representing the SEM. Adjusted *p* values generated from a one-way ANOVA with Tukey’s multiple comparisons test that were ≤0.05 are shown. (D) Stationary persisters are shown as the average of 10 independent experiments, with error bars representing the SEM. (E) Experimental design for treating stationary persisters formed in the presence or absence of loratadine followed by treatment with antibiotics and loratadine. Persisters formed in the presence of (F) ciprofloxacin, (G) vancomycin, or (H) gentamicin. In panels F–H, averages from three independent experiments are shown, with error bars representing the SEM.

We next measured a change that is known to occur in *S. aureus* persisters: lowered intracellular ATP levels.^43^ It has been established that bacterial persister formation depends on growth stage.^44^ MRSA cultures grown to stationary phase contain almost all persister cells.^45,46^ These cells have depleted intracellular ATP levels, which result in decreases in ATP-dependent antibiotic targets, rendering this subpopulation of bacteria tolerant to antibiotics.^43^ Therefore, there is an inverse relationship between persister formation and intracellular ATP levels. MRSA 43300 cultures were treated with loratadine or oxacillin or cotreated as described for RNA purification experiments (see Methods) to capture ATP levels when using the same conditions where ribosomal gene changes were detected. After 1 h of treatment, loratadine and cotreated cultures showed lower levels of intracellular ATP than the untreated control (Figure 7A). These levels fluctuated at 8 h of continuous treatment (Figure 7B). By 24 h of continuous treatment, we again observed lowered ATP levels whenever loratadine was present. At this time point, reductions were statistically significant (Figure 7C). We cannot make a direct correlation between ATP levels and persisters because ATP levels are influenced collectively by a number of metabolic processes, many of which we have shown loratadine to influence. That might explain why lowered levels of ATP were not detected in the oxacillin-treated cells, which would have been expected based solely on ribosomal gene modulation results (Figure 5A). Additionally, the ATP assay relies on viable cells, so decreased signal especially after 24 h of treatment could be more indicative of antibiotic potentiation in the cotreated bacteria. What we can conclude from this experiment is that loratadine and loratadine with oxacillin decrease intracellular ATP levels, consistent with our initial observation of reduction in ribosomal gene expression.

We wanted to more directly test the hypothesis that loratadine promotes persister formation in MRSA given the reduction in ribosomal gene expression and ATP. These persisters were enumerated in stationary phase cultures grown for 16 h with or without 50 μM loratadine. Loratadine treatment did not change the number of stationary persisters to a statistically significant level compared to untreated controls (*p* = 0.1771) (Figure 7D). This suggests that loratadine does not effectively prevent or enhance persister formation. To further enumerate persisters, we next exposed these stationary persisters to antibiotics at 10× their MIC for strain 43300. We observed the hallmark of persister formation, a biphasic kill curve (Figure S10). This drug-induced approach served to kill all nonpersisters from the population, resulting in less than 1% of the colony forming units (CFU) present compared to before antibiotic treatment (data not shown) (Figure 7E). Oxacillin was not used in this experiment because MRSA 43300 is resistant to it, and we have shown that loratadine potentiates this antibiotic in strain 43300.^7^ Therefore, to differentiate antibiotic resistance from antibiotic persistence, we tested three antibiotics other than oxacillin with different mechanisms of action. Ciprofloxacin, vancomycin, and gentamicin persisters did not significantly vary in number when comparing the control to loratadine-treated cultures (Figure 7F–H). Together, these results support loratadine decreasing intracellular ATP levels but not increasing persisters.

### Loratadine Treatment Prevents Biofilm Formation without Altering Cellular Morphology

We have previously reported that loratadine inhibits MRSA biofilm formation via nontoxic mechanisms. The average minimum biofilm inhibitory concentration (MBIC_50_) for strain ATCC 43300 was 11.49 μM.^7^ Accordingly, our RNA-seq results show that loratadine treatment upregulates several genes with ties to biofilm formation based on the UniProt keyword “biofilm” including *icaR*, *clpP*, *sarA*, and *traP* and downregulates *cshA* (Figure S8 and Table S4). Our previous reports relied on a standard crystal violet biofilm assay that allows for the quantification of biofilm mass but cannot provide information about the morphology of the biofilms or of the individual cells in the biofilm. We turned to scanning electron microscopy (SEM) to determine how loratadine treatment impacts these features of MRSA biofilms; 440× magnification provided a view of global biofilm morphology and topography, whereas 4000× magnification provided clearer images of the MRSA cells and their morphology.

**Figure 8:**
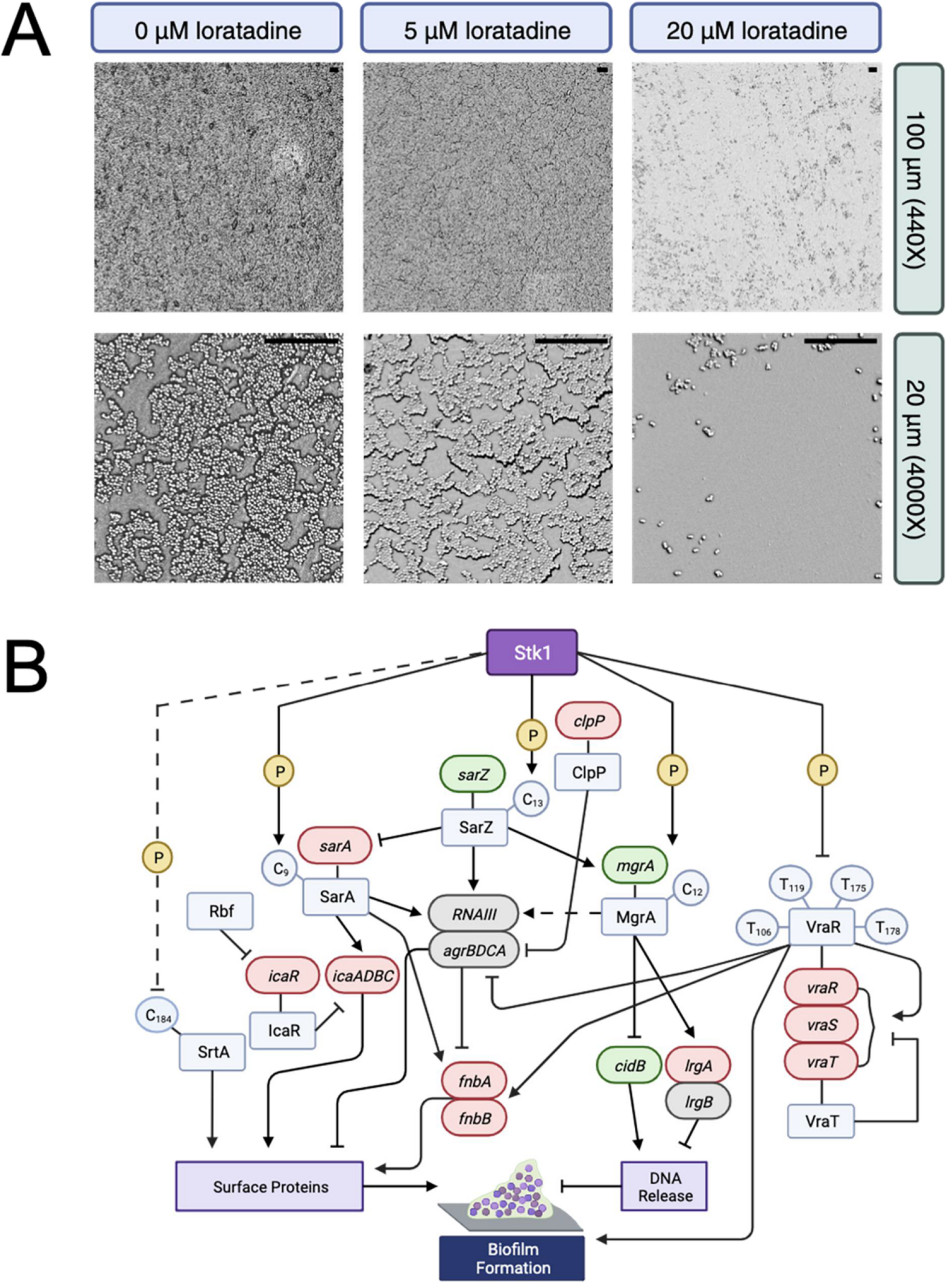
Loratadine treatment prevents MRSA biofilm formation**.** (A) MRSA ATCC 43300 biofilms were grown statically on PDMS in the presence or absence of loratadine at varying concentrations and imaged using scanning electron microscopy. Scale bars in the top right of each image represent 20 μm. (B) Key genes and proteins known to impact *S. aureus* biofilm formation are shown. Transcriptional changes with loratadine treatment as compared to untreated are indicated by gene shading. Genes that are downregulated are colored in green, genes that are upregulated are colored in red, and genes that show no significant change from untreated are in gray.

Untreated MRSA biofilms showed dense biofilm formation on a polydimethylsiloxane (PDMS) surface with evidence of towering and channels between biofilms (Figure 8A). At the cellular level, untreated MRSA cells were spherical and tightly packed. Loratadine treatment was examined at concentrations below and above the previously reported MBIC_50_.^7^ Cells treated with 5 μM loratadine showed minimal effects on biofilm or cellular morphology. Cells treated with 20 μM loratadine showed no notable changes in cellular morphology that would indicate that loratadine was disrupting the integrity of the cellular membrane or peptidoglycan. However, changes in the structure and density of the biofilm were readily evident. The biofilm clusters were much smaller and more diffuse across the PDMS surface, with wide swaths of empty space between the microcolonies. These results provide important evidence in favor of loratadine disrupting one or more critical signaling pathways necessary for biofilm formation. It is clear from our previous work that loratadine is not toxic to *S. aureus* at the tested concentrations,^7^ and the SEM images demonstrate that loratadine affects biofilm structures and densities without impacting cellular morphology. Figure 8B illustrates the many DEGs related to biofilms that may collectively contribute to the observed biofilm inhibition and further support loratadine inhibiting Stk1.

### Stk1 and Key Substrates Are Critical Regulatory Links between Biofilm Formation, Antibiotic Resistance, Virulence, and Metabolism in *S. aureus* and Are Modulated by Loratadine

Given that loratadine effectively disrupted biofilm formation *in vitro* and biofilms are intricately linked to both antibiotic resistance and virulence,^47−49^ it is not surprising that loratadine also changes gene expression in these additional arenas (Figures S11 and S12). Central metabolic pathways are also intricately connected to the virulence and resistance mechanisms *S. aureus* employs. Notably, CodY links central metabolic pathways to virulence^50−54^ and has recently been shown to work with catabolite control protein (CcpA) in a coordinated fashion to regulate these processes as well as biofilm formation.^55^ The mRNA levels for this global transcriptional regulator *codY* were significantly downregulated only with cotreatment (Figure S13). CcpA is a known Stk1 substrate, and phosphorylation inhibits its function.^56^ Therefore, in addition to decreasing transcript levels of *codY*, it is possible that loratadine also inhibits the phosphorylation of CcpA by Stk1, resulting in more active CcpA and the observed downregulation of *tst* and possibly other toxins (Figure S12). This modulation of CcpA and CodY is yet another example of how loratadine can influence both biofilm formation and antibiotic resistance (the two major phenotypic changes we previously reported).^7^

Stk1 phosphorylates the TCS member VraR.^16^ So, we examined the RNA-seq data for every *S. aureus* TCS member to determine additional “high-level” influence that Stk1 might exert that would be consistent with the widespread DEGs in interconnected pathways we had measured. We found that 11 TCS members were modulated by loratadine alone and 9 members were modulated by cotreatment. These TCSs primarily had regulatory functions surrounding biofilms, antibiotic resistance, cell wall synthesis, and survival and fitness. Interestingly, three genes that were upregulated with loratadine alone decreased in the presence of loratadine and oxacillin cotreatment: *lytR*, *vraS*, and *hssR* (Figure S14). Combined with other transcriptomic changes induced by loratadine through Stk1, those stemming from TCSs may help explain the number and diversity of genes that are affected with loratadine alone or in combination with oxacillin.

### Loratadine Is Not Toxic to Human Cells in Culture at Concentrations Used in Transcriptome Analysis

Although loratadine is FDA approved for use in children and adults as an antihistamine, its potential toxicity as an antibiotic adjuvant was next examined in a human cell culture with concentrations above and below what was used in our transcriptomic analysis (50 μM). Cell viability results are shown from a representative experiment in Figure S15. Compared to untreated control HEK 293 cells, only the highest concentration of loratadine tested (100 μM) showed significantly decreased viability. Following the treated cells’ viability for another 2 days (day 3 in Figure S15) showed similar results. The 50 μM treatment did have lower viability than the control but was significantly higher than the 100 μM treatment. Therefore, this result supports 50 μM loratadine eliciting widespread gene expression changes in MRSA, particularly disrupting metabolic processes, ribosomal genes, antibiotic resistance, and biofilm formation^7^ without increased toxicity to human cells in culture.

### Loratadine Enhances *C. elegans* Survival in an MRSA Infection Model

**Figure 9:**
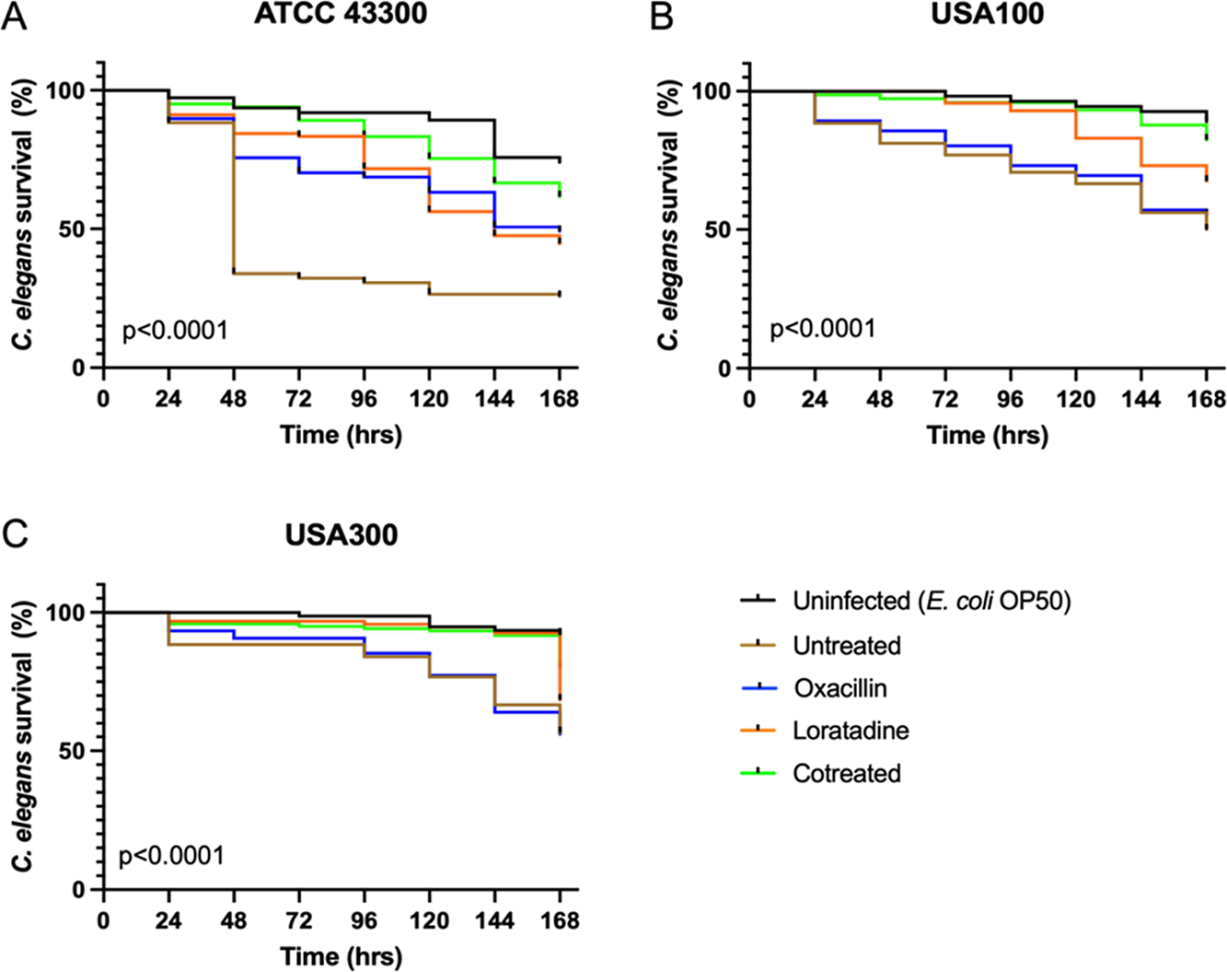
Loratadine enhances *C. elegans* survival in an MRSA infection model. (A) *C. elegans* exposed to the ATCC 43300 laboratory strain of MRSA, (B) hospital-acquired USA100 strain of MRSA, or (C) community-acquired USA300 strain of MRSA. All panels show one representative experiment. The reported adjusted *p* values come from log-rank (Mantel–Cox) tests.

We next wanted to examine loratadine treatment in an animal infection model of MRSA. The nematode *Caenorhabditis elegans* was chosen because of this organism’s short reproductive cycle, small size (amenable to multiple treatments and large sample size within one 96-well plate), and documented use in *S. aureus* infection studies.^57−61^*C. elegans* survival was monitored for 7 days after an uninfected control treatment (*E. coli* OP50 as food), MRSA 43300, oxacillin (4 μg mL^–1^), loratadine (50 μM), or both drugs in combination. As shown in Figure 9, the majority of uninfected *C. elegans* survived the entire time course, whereas untreated *C. elegans* quickly died, with a median survival time of only 48 h (Table S26). Either oxacillin or loratadine treatment alone significantly increased worm survival compared to untreated worms. Those worms had a median survival time of 168 and 144 h, respectively (Table S26). The highest survival enhancement was seen with cotreatment of oxacillin and loratadine. When testing these treatments with the USA100 hospital-acquired MRSA strain, we also observed significantly different *C. elegans* survival curves. Cotreatment still led to the highest survival, with the most significant enhancement compared to untreated *C. elegans* (*p* < 0.0001). Finally, the USA300 community-acquired MRSA strain was tested. Surprisingly, in this hypervirulent strain, loratadine treatment alone enhanced survival similarly to cotreatment (*p* = 0.07).

Consistent with our previous report, desloratadine (the active antihistamine metabolite of loratadine, marketed as Clarinex) did not act as an adjuvant in *C. elegans* infection assays (data not shown).^7^ Loratadine was dosed only once at the beginning of the assay and would be subject to metabolism to desloratadine by esterases in *C. elegans*.^62^ At this time, we cannot determine whether desloratadine is inactive in these assays because it does not engage with the same molecular target(s) as loratadine or if the carboxyethyl tail of loratadine is required for uptake into the bacterial cell. Studies are ongoing to determine the answers to these questions. Across all strains tested, loratadine alone enhanced survival over the untreated and oxacillin-treated controls. The results of the *C. elegans* infection assays suggest that loratadine may be an effective anti-infective treatment. Together, these data support loratadine extending animal survival upon MRSA infection by modulating the expression of MRSA genes critical for survival in a host. Loratadine as an adjuvant with oxacillin or loratadine alone may be sufficient for enhancing survival due to strain-specific differences in virulence gene expression. Additional experiments are ongoing to extend these findings to other medically relevant strains, explore pharmaceutically appropriate timing of loratadine doses, and explore more complex host models of infection.

## Discussion

**Figure 10:**
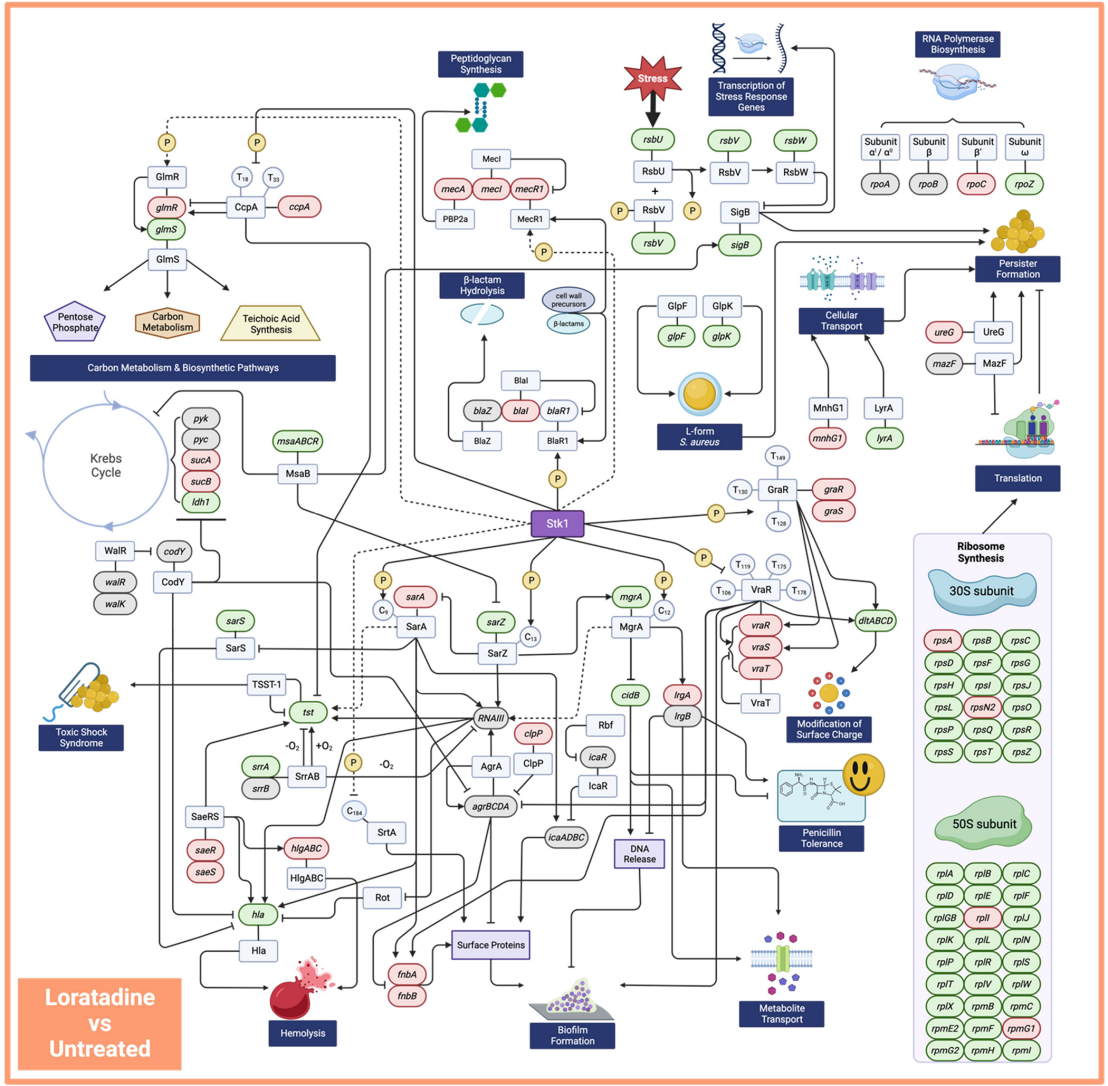
Summary of key genes and proteins known to impact *S. aureus* virulence, antibiotic resistance, and metabolism. Transcript-level changes with loratadine treatment as compared to no treatment are indicated by gene shading. Genes that are downregulated are colored in green, genes that are upregulated are colored in red, and genes that show no significant change from untreated are in gray. Solid lines represent published and verified interactions. Dashed lines represent hypothesized interactions that have not been experimentally verified. Although genes in the *bla* operon were not detected in our RNA-seq experiment, we have previously reported the effects of loratadine on these genes in ATCC 43300.^7^

**Figure 11:**
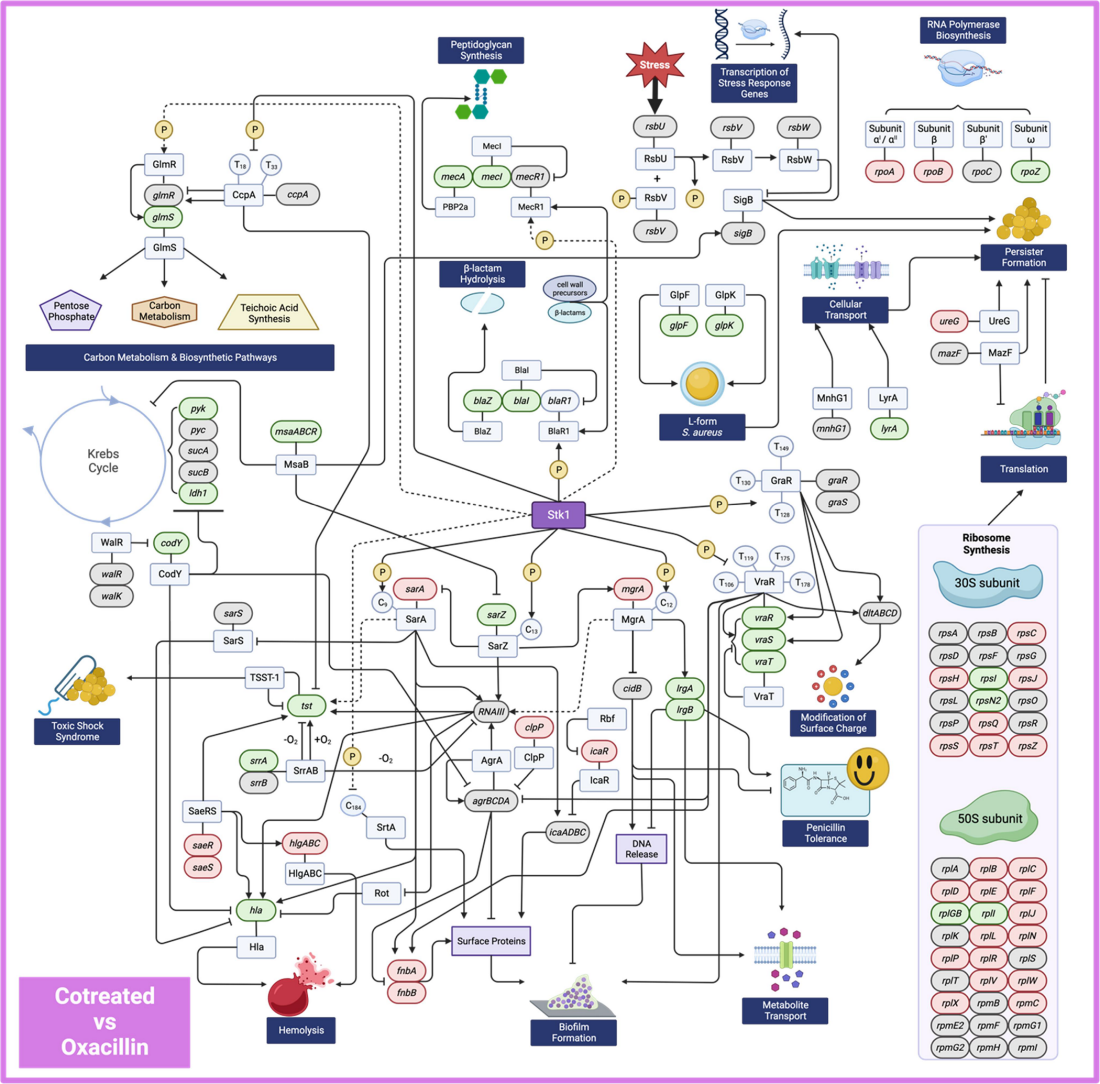
Summary of key genes and proteins known to impact *S. aureus* virulence, antibiotic resistance, and metabolism. Transcript-level changes with loratadine and oxacillin cotreatment as compared to oxacillin alone are indicated by gene shading. Genes that are downregulated are colored in green, genes that are upregulated are colored in red, and genes that show no significant change from untreated are in gray. Solid lines represent published and verified interactions. Dashed lines represent hypothesized interactions that have not been experimentally verified. Although genes in the *bla* operon were not detected in our RNA-seq experiment, we have previously reported the effects of loratadine on these genes in ATCC 43300.^7^

This study provides molecular details concerning oxacillin potentiation by loratadine in MRSA ATCC 43300. The multitude of transcript-level changes in virulence, antibiotic resistance, biofilm, metabolism, and the transcriptional and translational machinery genes in this pathogen position loratadine as an attractive anti-infective agent poised for further clinical study (Figures 10 and 11).

Consistent with our previous report,^7^ we showed that loratadine works as an antibiotic adjuvant, in part by modulating genes in the *mec* operon. Expanding on those molecular details, we have broadened our knowledge of other loratadine-controlled DEGs. The hypothesis that loratadine cotreatment would affect more than just a few key antibiotic resistance genes was supported. In fact, β-lactam resistance was not a significantly enriched pathway in this study. The observation that several biochemical pathways and several distinct, well-populated subclusters of DEGs were identified further emphasizes the utility of loratadine as an MRSA gene expression effector.

Because of their high-throughput nature, RNA-seq studies inherently include false positives and false negatives. For example, some genes that are likely affected by our drug treatments are missed. We did not detect RNA-seq read counts for genes in the *bla* operon, but we have shown that these genes are modulated by loratadine via RT-qPCR.^7^ Although this limitation exists for all RNA-seq studies, we have added confidence in our results through validation of sequencing efforts with RT-qPCR experiments (Figure 3). Another limitation is that the widely used STRING database is not an exhaustive list of interactions. There are more DEGs measured by RNA-seq than are in the database used to create interaction networks. Furthermore, some interactions are predicted and not experimentally validated. Others are based on homologous genes in other organisms. Therefore, we present transcriptome-wide effects caused by loratadine and oxacillin treatment, acknowledging that some effects are missed and others are included as a result of limited knowledge in *S. aureus*. We also created manually curated networks (Figures 10 and 11, Figures S9, S11–14, S16, and 17) that complement those based on the STRING database. These provide the following advantages: (1) They offer a targeted examination of our hypothesis that Stk1 is the likely molecular target of loratadine, and (2) they illustrate clinically relevant phenotypes that are important for examining loratadine as a potential therapeutic. By collecting gene and protein interacting data on Stk1, we have revealed extensive disruption in gene expression to many Stk1 targets. These disruptions include genes that are major players in antibiotic resistance and tolerance (Figure S11), toxins and virulence (Figure S12), and biofilms (Figure S16). Complementing the transcriptomic results with phenotypic assays such as persister formation (Figure 7), biofilm inhibition (Figure 8), and a host infection assay (Figure 9) allows us to consider the multidimensional gene expression effects of loratadine treatment on biological outcomes.

Although statistically enriched categories and pathways provide strong support of a cellular function being affected, individual or small numbers of genes can also greatly impact cellular processes when up- or downregulated. Of note, we detected *glmS* mRNA levels significantly downregulated with loratadine treatment and cotreatment (Tables S4 and S5). This gene encodes glutamine-fructose-6-phosphate aminotransferase and is critical for cell wall formation. It is considered the key “gatekeeper” of sugars, distributing them into either the glycolytic pathway or cell wall synthesis, but was not accompanied by enough other DEGs in the same pathway to constitute an enriched pathway. As *glmS* mutants showed enhanced susceptibility to oxacillin and other cell-wall active antibiotics,^63^ it is possible that lowered expression of this uniquely positioned gene contributes to the observed antibiotic potentiation. This connection is strengthened by results reporting that Stk1 stimulates expression of *glmS.*(64) Another example of a loratadine-modulated DEG on its own having widespread impact cell-wide is the biofilm regulator *icaR*. This gene product represses the translation of the *icaABDC* operon.^65^*icaR* mRNA levels were increased when cells were cotreated with loratadine and oxacillin (Figure S16). This transcript-level information is consistent with our phenotypic results demonstrating biofilm inhibition (Figure 8).

These results support loratadine inhibiting the master regulatory protein Stk1. For the first time, we report a loratadine-dependent downregulation of both *stk1* and its cognate phosphatase *stp1* (Tables S4 and S5). Using RT-qPCR, the subtle modulation in *stk1* mRNA levels was not detected in our previous analysis,^7^ nor was it in this report (Figure S3). Using RNA-seq, a more sensitive technique, we were able to detect subtle yet statistically significant modulation in both the kinase and the phosphatase genes. These two genes are transcribed from the same six gene operon.^66^ Accordingly, all six genes (*stk1, stp1, rlmN, rsmB, fmt,* and *def1*) are modulated similarly in our results (Tables S4–S9), but information concerning their transcriptional control is lacking. Importantly, genes that are cotranscribed in this operon have products with roles in translation (methyltransferases, formyltransferases, and a deformylase). The *rlmN* gene encodes a ubiquitous methyltransferase^67^ that may mediate resistance to antibiotics that function via inhibiting protein synthesis.^68^ Future experiments are needed to address this operon’s transcriptional regulation and contribution to antibiotic resistance directly.

One of the most widespread effects we detected was modulation of MRSA’s ribosomal genes and others involved in translation (Figures 5 and 6 and Table S24). Although we did not detect loratadine contributing to persister formation or maintenance, these disruptions to translation-related genes indicate that Stk1 inhibition by loratadine influences MRSA protein biosynthesis. Repression of translation occurs partly via phosphorylation of universally conserved elongation factor Tu (EF-Tu, encoded by the *tuf* gene in *S. aureus*).^69^ Stk1 has previously been reported to phosphorylate ribosomal proteins and EF-Tu in a phosphoproteomic analysis.^31^ Another group recently showed that Stk1 and Stp1, in particular, change the phosphorylation status of many ribosomal proteins and EF-Tu as a result of antibiotic and pH stress. When *stp1* was deleted from the Cowan I strain of *S. aureus*, the overall number of phosphopeptides in the cell increased, especially in ribosomal proteins and elongation factors. This led to a decrease in the overall protein synthesis and an increase in antibiotic tolerance compared to the wild-type strain.^70^ Although that report cannot be directly compared to ours (they examined differentially expressed proteins, not genes, and they used deletion mutants, not an inhibitory molecule like loratadine), the results are intriguing because of the high degree of overlap in affected target genes/proteins. We found that loratadine reduced *stp1* mRNA levels (Table S4 and Figure S3), and in those cells, the downregulated genes were overrepresented with ribosomal genes. Loratadine and oxacillin used in combination also reduced *stp1* mRNA levels (Table S5 and Figure S3) but created an uptick in ribosomal and *tuf* transcript levels toward untreated levels (Figure 5). *Tuf* transcript levels were uniquely modulated by loratadine and oxacillin cotreatment (no significant change in expression was detected with loratadine alone) (Figure 6). We propose that by regulating ribosomal genes and others like *tuf* that are key to translation, loratadine and oxacillin cotreatment partially counteracts that translational repression response in a multifaceted approach (Figures S9). This phenomenon could not have been detected if loratadine-treated cultures were only compared to an untreated control (Figure 1). We emphasize this experimental design because transcriptomic studies on antibiotic adjuvants often neglect to include the adjuvant molecule in combination with the antibiotic it potentiates.^9,71^

As striking as the widespread changes to the translational machinery were, we should also emphasize that MRSA’s transcriptional machinery was also significantly affected by loratadine. Every subunit of the core RNA Polymerase as well as several sigma factors like the alternative sigma factor B (*sigB*) had their mRNA levels significantly affected by loratadine (Figure S17 and Tables S4–S9). The mRNA levels of *sigB* were also identified by another group as being significantly modulated by loratadine.^11^ Adjusting the gene expression of these transcriptional players and stress-related sigma factors themselves would be expected to make a huge impact on MRSA gene expression. Although multiple pathways are being modulated, likely through Stk1, this is not due to nonspecific binding of a small molecule. There are many distinctions between the DEGs measured by our group with loratadine compared to the much smaller 4-bromocarbazole, for example.^72^ The categories of DEGs modulated by loratadine illustrate significant, biologically relevant, and yet widespread changes.

A previous report on loratadine as an antibiotic (vancomycin) adjuvant in MRSA showed that the molecule likely disrupts the interaction between Stk1 and MgrA by binding MgrA directly.^11^ Both Stk1 and Stp1 act on MgrA as a substrate.^29^ We also found that *mgrA* mRNA levels were significantly reduced whenever loratadine was present (Tables S4 and S5). Although we have not yet demonstrated a direct interaction *in vitro* between loratadine and Stk1 or Stp1, it is possible that these three proteins are some of the top-level global regulators impacting loratadine effects cell wide (Figures 10 and 11)

We are the first to report loratadine extending *C. elegans* survival upon MRSA infection. This data also confirmed that loratadine alone was not toxic to the nematodes using the same concentrations used in our transcriptome analysis. In the *C. elegans* infection model, we reported reduced mortality after MRSA infection upon loratadine and oxacillin cotreatment compared to a control. This was most prominent in the ATCC 43300 strain and, to a lesser extent, hospital-acquired USA100 and community-acquired USA300 strains. Loratadine alone also showed reduced mortality compared to untreated infected worms (Figure 9 and Table S26). These experimental results also agree with Zheng et al, who showed that loratadine treatment alone reduced the mortality of mice that had a pulmonary *S. aureus* infection.^11^ Together, these studies point to the need for more research on loratadine’s potential clinical benefits in MRSA infection models.

This transcriptomic study was performed in strain ATCC 43300. It is a widely used reference strain of HA-MRSA. Because of strain-specific differences in antibiotic potentiation and transcriptional regulation,^7^ it will be critical to expand RNA-seq studies to other strains. Although the *C. elegans* infection model data reported here support loratadine functioning effectively in multiple strains (Figure 9), analysis of clinically relevant strains (both hospital- and community-acquired) of various SCCmec types will paint a more thorough picture of loratadine’s role as an antibiotic adjuvant and its potential utility as a novel treatment for *S. aureus* infections.

## Methods

### MRSA Cultures and Treatment for RNA Purification

*S. aureus* 43300 were purchased from the American Type Culture Collection (ATCC). Cultures were grown overnight in cation-adjusted Mueller–Hinton Broth (CAMHB) at 37 °C with shaking. Mid log-phase cultures were diluted to 5 × 10^5^ CFU mL^–1^ in CAMHB. The cell suspension was aliquoted into sterile culture tubes, and loratadine was added to a final concentration of 50 μM (<25% of the compound’s MIC). Oxacillin was added to a separate culture tube at a final concentration of 4 μg mL^–1^ (<25% of its MIC). Cotreatment consisted of both oxacillin (4 μg mL^–1^) and loratadine (50 μM). Cultures treated with solvent DMSO served as negative controls. Each of the four conditions was performed in triplicate. Cultures were incubated at 37 °C with shaking for 1 h. After treatment, cells were pelleted and placed in a −80 °C freezer until total RNA purification.

### Total RNA Purification

Total RNA was purified using a Qiagen RNeasy kit as previously described.^73^ RNA concentration and purity were determined using a Nanodrop microvolume spectrophotometer.

### RNA-seq

All subsequent RNA-seq steps, including additional RNA quality control (Supporting Information Table S1), library construction, sequencing (Supporting Information Table S2), and reference genome alignment (Supporting Information Table S3), were conducted by Novogene, Inc., as reported previously.^74^ Experimental details and raw and processed data were deposited to Gene Expression Omnibus (GEO) under accession number GSE227099.

Triplicate samples in each treatment group were analyzed originally, but 1 of the 12 samples did not meet the recommended 0.92 Pearson correlation value to be included as part of the same replicate group. One cotreated sample (Co_A) had a Pearson correlation value of 0.875 with Co_B and 0.897 with Co_C (Supporting Information Figures S1 and S2). This individual sample was excluded from all subsequent analyses.

### Bioinformatics

Bioinformatic analyses were conducted by Novogene, Inc. This included novel gene detection, quantification, differential gene expression, and Gene Ontology (GO)^75^ and Kyoto Encyclopedia of Genes and Genomes (KEGG)^76^ enrichment. Resulting GO categories completely contained within others were checked for ancestry using directed acyclic graphs. Only the most specific child categories were reported. The Venn diagram was created using the multiple lists comparator tool at https://molbiotools.com/listcompare.php.

### RT-qPCR

RNA purification was performed on four biological replicates, independent from those used in RNA-seq analysis as described previously.^73^ Reverse transcription and polymerase chain reaction were performed as described previously.^73^ All primer information can be found in Table S10. Data were graphed and analyzed for statistical significance using a one-way ANOVA with Tukey’s multiple comparisons test in GraphPad Prism.

### Network Analysis

Interaction networks were constructed using the STRING database in Cytoscape v 3.9.1^77^ as previously reported.^74^ Additionally, interaction networks were formed from each subcluster of DEGs. In all cases, individual nodes with no edges were removed from the figures. The statistical significance of interaction enrichment was calculated using Cytoscape’s Functional Enrichment tool, with the *S. aureus* genome used as background. The MCODE^78^ and cytoHubba^79^ apps were used to help identify hub nodes. cytoHubba sorted nodes by maximal clique centrality (MCC).^79^ The full networks are available for interactive visualization in Cytoscape by accessing them on NDex as shown in Supporting Information Figures S7, S8, and 6.

### ATP Assays

ATCC 43300 cells were grown and treated with loratadine and/or oxacillin as described above. At 1, 8, or 24 h of incubation, an aliquot was removed for use in the Promega BacTiter Glo Assay according to manufacturer’s instructions. After washing the cells, they were normalized to the same OD_600_ (the lowest value achieved by the four treated samples) in PBS. Technical triplicates with a volume of 100 μL each were added to white 96-well plates. PBS-containing wells served as background. Luminescence was measured in a BioTek Syngergy plate reader. Four separate experiments were performed per time point so that luminescence values were background corrected and averaged between four biological replicates. Each treatment’s normalized luminescence is reported relative to the untreated control. Data were graphed and analyzed using GraphPad Prism. Error bars show the standard error of the mean, and statistical significance was determined using a one-way ANOVA with Dunnett’s multiple comparisons test.

### Persister Assays

ATCC 43300 cells were grown in CAMHB to stationary phase by incubating with shaking at 300 rpm at 37 °C for 16 h. Loratadine was either included at 50 μM final concentration or not included. Serial dilutions were made for spot plating on tryptic soy agar (TSA). Plates were incubated at 37 °C for 16 h, and CFU mL^–1^ were calculated. Ten biological replicates were performed on independent days. GraphPad Prism was used to calculate averages and standard error of the means and apply an unpaired *t* test for statistical significance. To enumerate drug-induced persisters, stationary cultures were grown as described above and then pelleted and washed with PBS twice. Each culture was then normalized to an OD_600_ of 0.4 before treating with ciprofloxacin (10 μg mL^–1^), gentamicin (640 μg mL^–1^), or vancomycin (20 μg mL^–1^), which represents experimentally determined 10× MICs for this strain. Parallel cultures with each antibiotic were also cotreated with loratadine (50 μM). These were incubated with shaking at 300 rpm at 37 °C for 4 h. Finally, serial dilutions were made for spot plating on TSA. Plates were incubated at 37 °C for 16 h, and CFU mL^–1^ were calculated. Three biological replicate experiments were performed. GraphPad Prism was used to calculate averages, standard error of the means, and a one-way ANOVA with Tukey’s multiple comparison test.

### Synthesis of PDMS Surfaces for Biofilm SEM

Polydimethylsiloxane (PDMS) was synthesized using a 10:1 ratio of base to curing agent (Sylgard 184). The resulting viscous liquid (300 mL) was added to each well of a 48-well plate made of polystyrene. PDMS was cured at 92 °C for 1 h. The plates were plasma cleaned for 1 min. Prior to use, plates were incubated in a sterile biosafety cabinet under UV light for ∼30 min to remove potential contamination.

### Biofilm Growth on PDMS Surfaces

A single colony of ATCC 43300 was used to inoculate 5 mL of TSBG and incubated with shaking (200 rpm) at 37 °C overnight. The overnight culture was diluted with fresh TSBG to an adjusted OD_600_ of 0.1. The bacterial suspension was split into aliquots of 25 mL. One aliquot was left untreated to serve as the negative control. The other aliquots were dosed with a stock solution of loratadine (25 mM in DMSO) to achieve the desired final loratadine concentrations. These aliquots were added to a 48-well plate containing purified polydimethylsiloxane (PDMS) prepared as described. Sterile deionized water (200 μL) was added to each well in columns 1 and 8. The untreated suspension (200 μL per well) was added to column 2 as the negative control. The other columns were filled with the loratadine-treated suspensions (200 μL). The plate was wrapped in Press-n-Seal and incubated at 37 °C for 24 h. Media and any planktonic bacteria were carefully removed from each well with a micropipette, taking care not to disturb any biofilms. The remaining planktonic cells were removed by addition of 0.1 M PBS (200 μL) and subsequent removal.

### Preparation of Biofilms for SEM Imaging

Biofilm samples were fixed in 2% paraformaldehyde (200 μL) overnight, rinsed in water and 0.1 M PBS, and dehydrated via a graded series of ethanol (50, 70, 90, and 100%). The samples were then treated with hexamethyldisilazane (HMDS) twice for 5 min each and air-dried for 15–25 min. A 5 mm size biopsy punch was used to collect specimens of PDMS with or without biofilms for SEM imaging. The specimens were sputter coated with gold and imaged using a Phenom XL scanning electron microscope.

### Human Cell Viability Assays

Human embryonic kidney cells (HEK 293s) were purchased from ATCC and cultured in Dulbecco’s modified Eagle’s medium (DMEM) with 10% fetal bovine serum and no antibiotics. Cultures were kept at 37 °C and 5% CO_2_ and were determined to be free of mycoplasma contamination via a qPCR assay. Viability assay plates (96 wells) contained 20,000 cells per well that were allowed to adhere overnight. Additional wells contained the medium only as background. All conditions were performed with six technical replicates. The next day, cells were treated with loratadine for a final concentration of 12.5, 25, 50, or 100 μM loratadine in DMSO (final concentration 0.1%). Untreated cells contained DMSO only (final concentration 0.1%). Treatment lasted for 24 h, and then the drug-containing medium was replaced with fresh DMEM supplemented with alamarBlue (Molecular Probes). Cells were incubated for 2 h, and then fluorescence was measured according to the manufacturer’s instructions. These results are shown as day 1 values. This 24 h treatment was repeated on a separate plate to allow viability to be measured after 2 days of growth following drug removal. These results are shown as day 3 values. Fluorescence was background corrected, and the average value was plotted. GraphPad Prism was used to calculate the mean and standard error of the mean and to perform a two-way ANOVA with Tukey’s multiple comparisons test.

### *C. elegans* Liquid Survival Assay

The *C. elegans* mutant strain SS104 was purchased from the Caenorhabditis Genetics Centre and was propagated and synchronized as previously reported.^57^ Approximately 10–20 worms were added per well of a 96-well plate. Each control and experimental treatment had six replicate wells (*n* ∼60–120 worms per treatment). *E. coli* OP50 culture was added as an uninfected control. *S. aureus* ATCC 43300, USA100, USA300, and COL were purchased from ATCC. Cultures were grown in tryptic soy broth (TSB) at 37 °C shaking overnight. They were normalized to an OD_600_ of 1.2 and added to the worms as infected treatments. Loratadine was used at a final concentration of 50 μM and oxacillin was used at a final concentration of 4 μg mL^–1^ to be consistent with RNA-seq and RT-qPCR experiments. The plate was kept at room temperature on a rocking platform for 7 days. Dead worms were scored every 24 h. Kaplan–Meier survival curves and median survival times were generated using GraphPad Prism. Statistically significant differences (*p* < 0.05) between survival curves were determined using the log-rank (Mantel–Cox) test within Prism. Four independent experiments were conducted for each strain of MRSA, with a representative experiment shown.

## Supporting Information Available

RNA-seq quality control results, RT-qPCR primer information, hub gene analysis, persister gene analysis, *C. elegans* median survival results, RNA-seq sample correlations, *stk1* and *stp1* RT-qPCR results, select KEGG pathway maps, STRING protein interaction networks, persister kill curve, human cell viability results, and networks involving genes related to persisters and translation, antibiotic resistance and tolerance, toxins and virulence, metabolism, two component systems, biofilms, and transcriptional regulation (PDF)
Complete differential gene expression (XLSX)
Gene Ontology and KEGG pathway results (XLSX)

## Notes

All illustrations were created with BioRender.com.

## Notes

This work involved biohazardous materials. All experiments involving bacteria were conducted according to BSL-2 guidelines.
