# Supplementary material for: Loratadine Combats Methicillin-Resistant Staphylococcus aureus by Modulating Virulence, Antibiotic Resistance, and Biofilm Genes
Source: ACS Infect Dis. 2023 Dec 28;10(1):232–50. doi: 10.1021/acsinfecdis.3c00616 (PMC10788911; doi:10.1021/acsinfecdis.3c00616)
Supplement: Supplementary file 1 — id3c00616_si_001.pdf [file id3c00616_si_001.pdf]

Supporting Information

Loratadine Combats Methicillin-Resistant *Staphylococcus aureus* by Modulating Virulence, Antibiotic Resistance, and Biofilm Genes

Brianna L. Viering[a], Halie Balogh[a], Chloe F. Cox[a], Owee K. Kirpekar[a], A. Luke Akers[a], Victoria A. Federico[b], Gabriel Z. Valenzano[a], Robin Stempel[a], Hannah L. Pickett[b], Pamela M. Lundin[a], Meghan S. Blackledge[a]*, Heather B. Miller[a]*

Author Affiliations:

[a]Department of Chemistry, High Point University, High Point, North Carolina 27268, United States

[b]Department of Biology, High Point University, High Point, North Carolina 27268, United States

*Corresponding Authors: Meghan S. Blackledge mblackle@highpoint.edu, Heather B. Miller hmiller@highpoint.edu

Preprint Server: An earlier version of this manuscript was deposited to ChemRxiv on March 23, 2023: https://doi.org/10.26434/chemrxiv-2023-zmt04. The content is available under CC BY NC ND 4.0.

Keywords: *S. aureus*, MRSA, antibiotic adjuvant, Stk1, loratadine

**Supporting Tables**	**Page**
S1 – Additional quality control details of RNA samples	S3
S2 – RNA-seq read quality control	S4
S3 – Mapped reads summary	S5
S4-S9 – contain complete DEG data sets and are available in an Excel workbook	
S10 – RT-qPCR primer information	S6
S11-S23 – contain completed GO and KEGG results and are available in an Excel workbook	
S24 – Hub analysis of genes affected by cotreatment but not loratadine alone	S7
S25 – DEGs related to persisters	S8
S26– Loratadine enhanced *C. elegans* survival when infected with MRSA	S9

Supporting Figures **Page**

Supporting Figures	Page
S1 – RNA sample Pearson correlation results	S10
S2 – Principal component analysis results	S11
S3 - *stk1* and *stp1* RT-qPCR analysis	S12
S4-S6 – KEGG pathway maps enriched with DEGs	S13-15
S7- S8 – STRING interaction networks	S16-17
S9 – DEGs related to persisters and translation	S18-19
S10 – Biphasic kill curve	S20
S11 – DEGs involved in antibiotic resistance and tolerance	S21-22
S12 – DEGs involved in toxins and virulence	S23-24
S13 – DEGs involved in metabolism	S25-26
S14 – DEGs that are two component systems (TCSs)	S27-28
S15– Human cell viability results	S29
S16 – DEGs that are involved in biofilms	S30-31
S17 – DEGs that are involved in transcriptional regulation	S32
References	S33-35

Supporting Tables

Supporting Table S1: RNA sample quality control summary. Un represents untreated sample, ox represents oxacillin treated sample, lor represents loratadine treated sample, and co represents cotreated sample. Each biological replicate is labelled A, B, or C. RIN = RNA integrity number

Sample #	Sample ID	Customer Sample ID	Sample Type	Sample Volume ul	Concentration ng/ul	Total Quantity ng	RIN
1	1735R-1563-01	un_A	RNA	30	136.00	4080	9.2
2	1735R-1563-02	ox_A	RNA	30	25.20	756	8.4
3	1735R-1563-03	lor_A	RNA	30	157.00	4710	9.0
4	1735R-1563-04	co_A	RNA	30	21.60	648	8.6
5	1735R-1563-05	un_B	RNA	30	223.00	6690	9.0
6	1735R-1563-06	ox_B	RNA	30	133.00	3990	8.8
7	1735R-1563-07	lor_B	RNA	30	67.40	2022	8.6
8	1735R-1563-08	co_B	RNA	30	150.00	4500	9.0
9	1735R-1563-09	un_C	RNA	30	328.00	9840	9.1
10	1735R-1563-10	ox_C	RNA	30	125.00	3750	8.9
11	1735R-1563-11	lor_C	RNA	30	250.00	7500	8.8
12	1735R-1563-12	co_C	RNA	30	220.00	6600	8.8

Supporting Table S2: RNA-seq reads quality control summary. Un represents untreated sample, ox represents oxacillin treated sample, Lor represents loratadine treated sample, and co represents cotreated sample. Each biological replicate is labelled A, B, or C. Q20 and Q30 were calculated as the base number of Phred value > 20 or 30, respectively, divided by the total base value x 100%.

Sample name	Raw reads	Clean reads	Raw bases G	Clean bases G	Error rate %	Q20 %	Q30 %	GC content %
Un_A	15267392	12540594	2.3G	1.89G	0.03	97.75	93.34	36.02
Ox_A	15814632	13515734	2.38G	2.03G	0.03	97.87	93.68	31.74
Lor_A	15070926	12788262	2.27G	1.92G	0.03	97.7	93.16	35.12
Co_A	14249984	12520916	2.14G	1.88G	0.03	97.87	93.66	30.65
Un_B	15783172	13624762	2.37G	2.05G	0.03	97.75	93.27	35.6
Ox_B	15351340	13234730	2.31G	1.99G	0.03	97.82	93.44	33.18
Lor_B	13976528	11734974	2.1G	1.77G	0.03	97.73	93.28	35.08
Co_B	15834374	13698268	2.38G	2.06G	0.03	97.83	93.44	34.29
Un_C	15118628	12896152	2.27G	1.94G	0.03	97.79	93.35	35.49
Ox_C	15460962	13054814	2.32G	1.96G	0.03	97.9	93.63	33.2
Lor_C	15341634	13149662	2.31G	1.98G	0.03	97.68	93.09	34.64
Co_C	15222306	13061784	2.29G	1.96G	0.03	97.86	93.48	34.13

Supporting Table S3: Mapped reads summary. Un represents untreated sample, Lor represents loratadine treated sample, Ox represents oxacillin treated sample, and Co represents cotreated sample. Each biological replicate is labelled A, B, or C.

**Sample name**	Co_A	Co_B	Co_C	Lor_A	Lor_B	Lor_C	Ox_A	Ox_B	Ox_C	Un_A	Un_B	Un_C
Total reads	12520916	13698268	13061784	12788262	11734974	13149662	13515734	13234730	13054814	12540594	13624762	12896152
Total mapped (%)	99.19	99.22	99.34	99.37	99.23	99.32	99.17	99.27	99.3	98.29	99.14	99.35
Multiple mapped (%)	3.04	5.62	3.57	5.01	5.73	3.75	4	5.87	3.54	6.23	6.46	6.09
Uniquely mapped (%)	96.15	93.6	95.77	94.37	93.5	95.58	95.17	93.4	95.76	92.06	92.68	93.27
Read-1 (%)	48.12	46.82	47.91	47.2	46.78	47.8	47.6	46.72	47.9	46.09	46.35	46.64
Read-2 (%)	48.03	46.79	47.87	47.16	46.72	47.77	47.54	46.68	47.87	45.97	46.33	46.63
Reads map to '+' (%)	48.04	46.	47.88	47.18	46.75	47.79	47.55	46.69	47.87	46.02	46.33	46.63
Reads map to '-' (%)	48.11	46.8	47.89	47.18	46.75	47.79	47.62	46.71	47.89	46.05	46.34	46.63
Reads mapped in proper pairs (%)	90.13	88.47	90.28	88.86	87.97	90.02	89.03	88.33	90.03	86.21	87.42	87.74
Proper-paired reads map to different chrom (%)	0	0	0	0	0	0	0	0	0	0	0	0

Supporting Tables S4-S9 – contain complete DEG data sets and are available in an Excel workbook

Supporting Table S10: RT-qPCR Primers. Primer sequences are written 5' to 3'.

gene	Forward primer sequence	Reverse primer sequence	Efficiency	Reference
lrgA	GACGCATCAAAACCAGC ACACTTT	CCGATTACTGATGCAGGCATA GGA	100.0%	Viering et al[1]
lrgB	AGCAGTTGGTTTAATCG CCCCTTT	ACAAAGACAGGCACAACTGC	80.3%	Viering et al[1]
mcsA	CGTCCGCAGACTTCAAG GTGG	TGTGGTGTCTTTCCAACGTGCT C	95.3 %	This work
ulaA	GCGGGCGTTAGTTATGT ACACGAATC	CACCAGCAGCAAATGTCACAC CT	104.5%	This work
ydfJ	GCAGTGGGAACAGCAG GTAGC	CCGCATACGGCAATCACAACT GT	91.9%	This work
sRNA 00031	ATTAATGCATCCCCGTG C	CGCATATTAATTAGGAGATGA AAGG	94.5%	This work
stk1	TGTGGCAATGGCAATGT TTGG	ACCAAACATTGCCATTGCCAC	91.9%	This work
stp1	GCCATAAAGCAGGAGA AGTTGCAAG	TCCGCTTCAAAACGGGATTTC A	107.0%	This work
16S	CTGTGCACATCTTGACG GTA	TCAGCGTCAGTTACAGAC CA	93.8%	Yarwood et al[2]

Supporting Tables S11-S23 – contain completed GO and KEGG results and are available in an Excel workbook

Supporting Table S24: Hub analysis of genes affected by cotreatment but not loratadine alone. Rank order and score was generated by the cytoHubba app within Cytoscape. DGE = differential gene expression. Stk1 interaction was determined via the STRING database and published literature.

Rank	Gene name	Description	Score	DGE	Known stk1 interactor?
1	*rpoB*	DNA-directed RNA Polymerase subunit beta	269	up	
2	*rpoA*	DNA-directed RNA Polymerase subunit alpha	267	up	
2	*rpsE*	30S ribosomal protein S5	267	up	
4	*rpmJ*	50S ribosomal protein L21	243	up	
4	*rplU*	50S ribosomal protein L36	243	down	
6	*tuf*	Elongation Factor Tu	146	down	yes
7	*rpsU*	30S ribosomal protein S21	122	up	
8	*cysS*	Cysteine - tRNA ligase	28	up	
9	*pyk*	Pyruvate kinase	8	up	yes
10	*birA*	Bifunctional ligase/repressor	7	up	

Supporting Table S25: DEGs Related to *S. aureus* Persisters

Gene Symbol	Description	Lor vs Un	Ox vs Un	Co vs Un
mazF[3]	Endoribonuclease MazF	ns	-0.8669522	-0.4832961
glpF[4]	Glycerol uptake facilitator protein	-1.3007258	ns	-1.6656859
glpK[4]	Glycerol kinase	-0.6153474	0.51204046	-0.6946478
lyrA (HOU_2315)[5]	Lysostaphin resistance protein A	-1.8559204	-1.1991132	-2.4184495
mnhG1[5]	Monovalent cation H^+ antiporter subunit G	1.04240713	0.64107148	0.99559515
ureG[5]	Urease accessory protein UreG	1.87279444	1.28268499	2.16658464
ctaB[5]	Protoheme IX farnesyltransferase	-1.5892108	-1.4116474	-2.3078889
malG (HOU_0223)[5]	Maltose ABC transporter permease	-0.4704239	-0.7942888	-1.2131888
fbaA[5]	Fructose-bisphosphate aldolase	ns	ns	ns
clpX[5]	ATP-dependent protease ATP-binding subunit ClpX	ns	ns	ns
parE[5]	DNA topoisomerase IV, B subunit	ns	-0.5373953	ns
purN[6]	Phosphoribosylglycinamide formyltransferase	-2.9778379	-2.0235189	-3.3778506
purF[7, 8]	Amidophosphoribosyltransferase	-2.9166569	-2.0919616	-3.1599823
purB[7, 8]	Adenylosuccinate lyase	-1.2717564	0.34674123	-0.4947821
purM[7, 8]	Phosphoribosylformylglycinamidine cyclo-ligase	-2.8449126	-2.1286574	-2.9815223
argJ[9]	Arginine biosynthesis bifunctional protein ArgJ	-1.2131515	ns	-1.0449835
phoU[10]	Phosphate-specific transport system accessory protein	ns	ns	ns
ctaB[11]	Protoheme IX farnesyltransferase 2	-1.5892108	-1.4116474	-2.3078889
*msaB (cspA)**[12]	Cold shock protein A	-1.5887983<br>-2.253419	-0.6049145<br>-2.7731155	-1.8279351<br>-3.7086833

Lor, loratadine; Ox, oxacillin; Co, cotreatment; Log2 fold changes are shown for each treatment comparison if padj ≤0.05; ns, not significant; *gene found twice in ATCC 43300 reference genome

Supporting Table S26: Loratadine enhanced *C. elegans* survival when infected with MRSA. Oxacillin was used at 4μg/mL and loratadine was used at a final concentration of 50μM . Cotreated wells contained both oxacillin at 4μg/mL and loratadine at 50μM. The number of hours where at least 50% of *C. elegans* survived is shown. Undefined is reported when at the end of the time course experiment, over 50% of *C. elegans* are still surviving and cannot be scored as dead.

	**Median Survival (hrs)**		
**Treatment**	**43300**	**USA100**	**USA300**
Uninfected (OP50)	Undefined	Undefined	Undefined
Untreated (MRSA)	48	168	Undefined
Oxacillin	168	Undefined	Undefined
Loratadine	144	Undefined	Undefined
Cotreated	Undefined	Undefined	Undefined

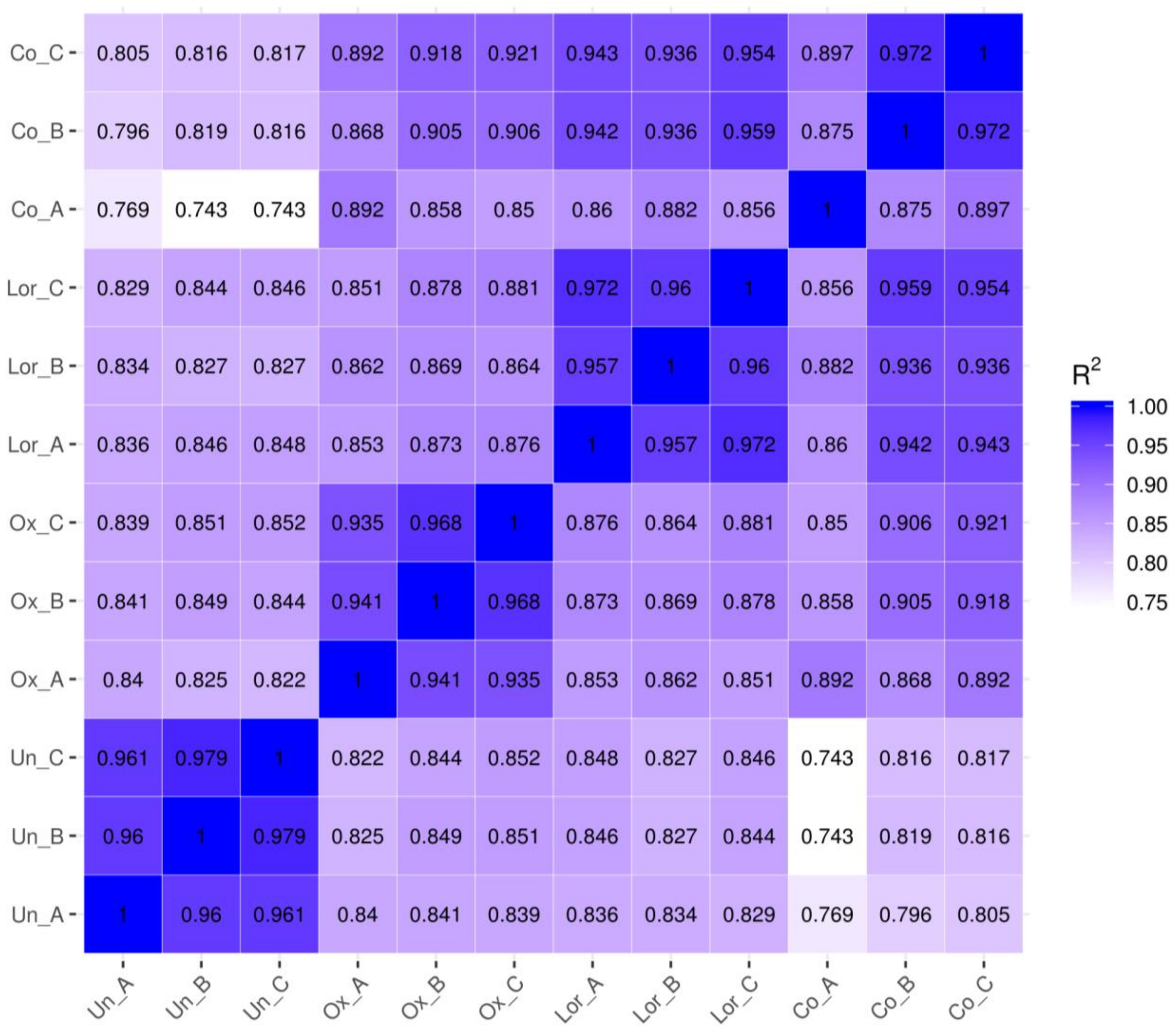

Supporting Figure S1: Pearson correlation between samples. A Pearson correlation coefficient was calculated for each pairwise comparison among the 12 RNA samples. Un represents untreated sample, Lor represents loratadine treated sample, Ox represents oxacillin treated sample, and Co represents cotreated sample. Each biological replicate is labelled A, B, or C.

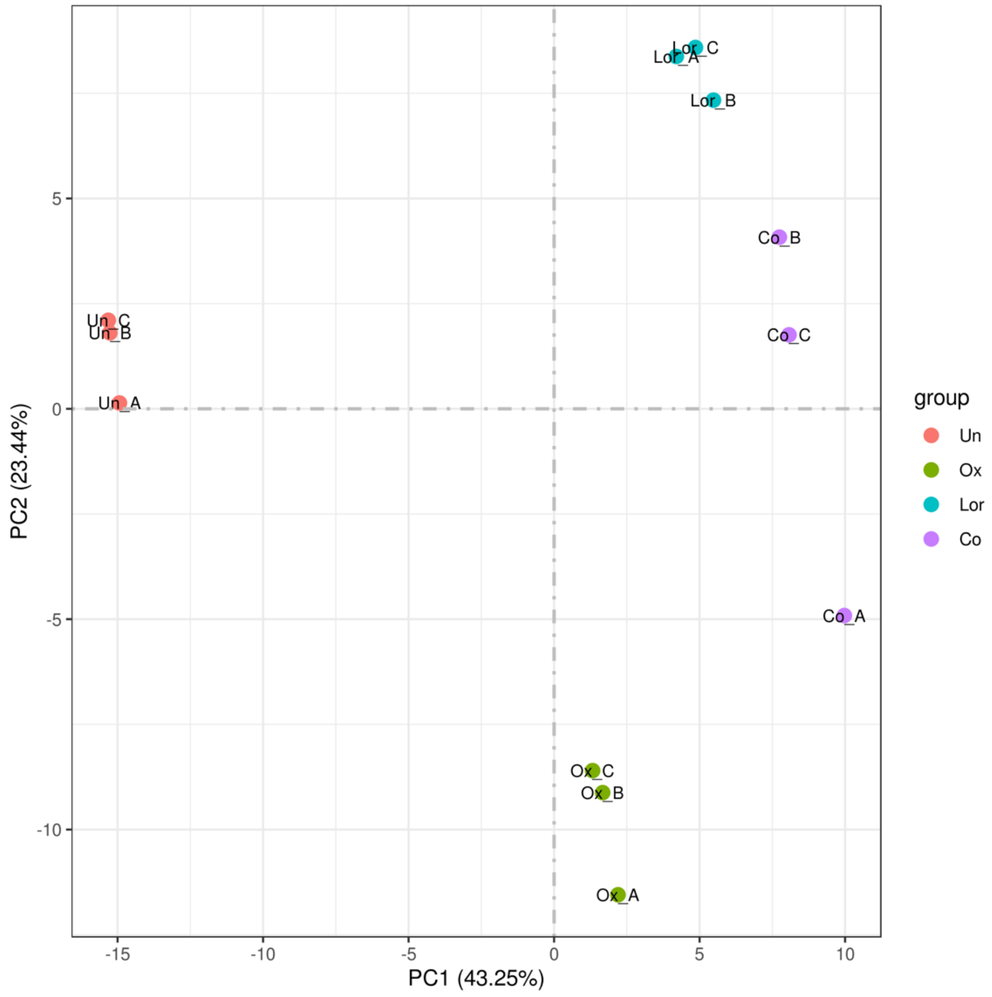

Supporting Figure S2: A Principal component analysis PCA was performed on the 12 RNA samples. Un represents untreated sample, Lor represents loratadine treated sample, Ox represents oxacillin treated sample, and Co represents cotreated sample. Each biological replicate is labelled A, B, or C.

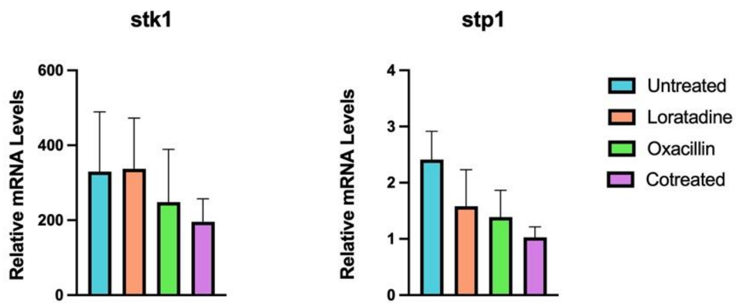

Supporting Figure S3: *stk1* and *stp1* mRNA levels were subtly altered by loratadine and/or oxacillin, as measured by RT-qPCR. The y axis displays the average mRNA levels relative to *16S* rRNA. Error bars represent the standard error of the mean.

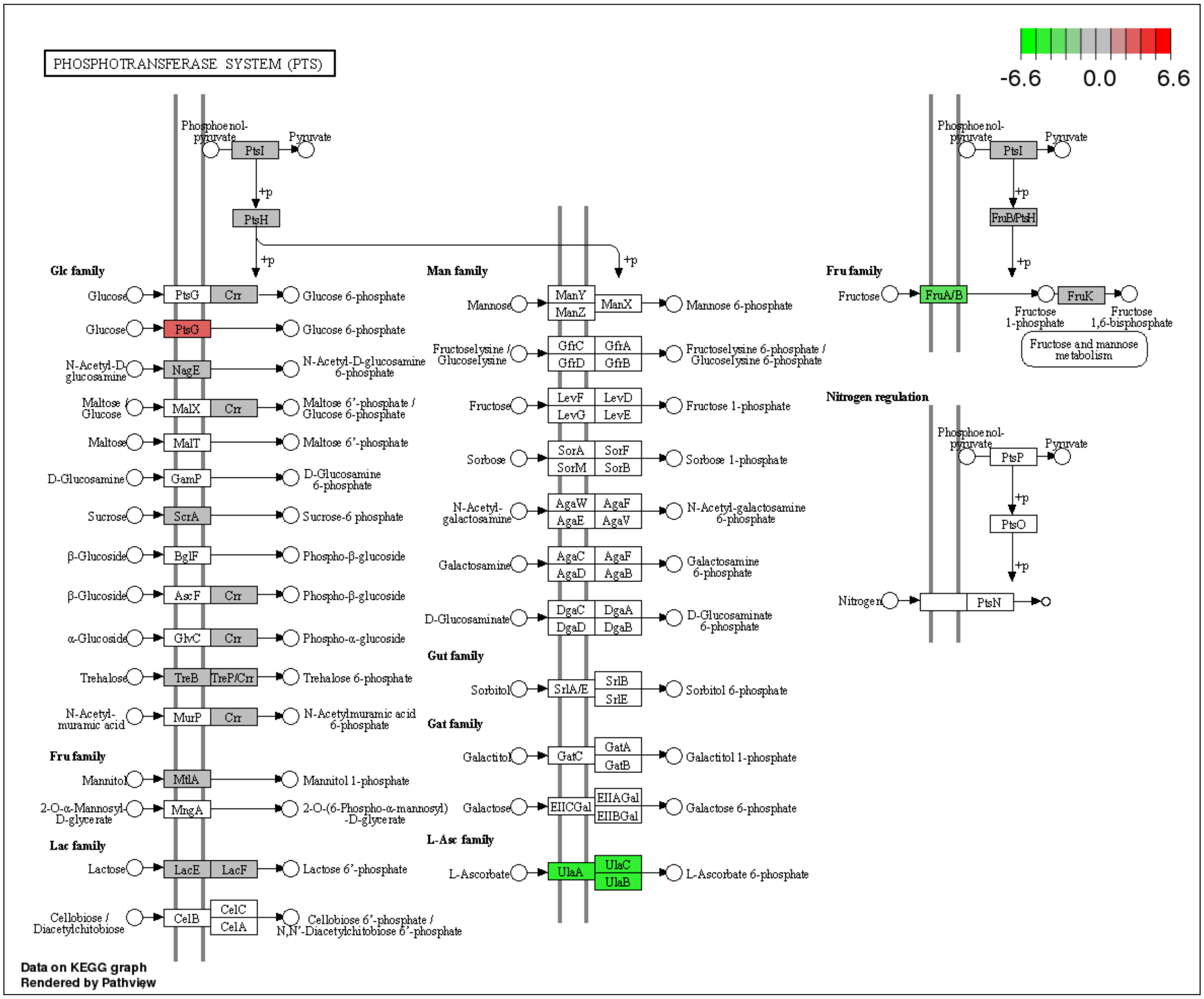

Supporting Figure S4: KEGG pathway map of the phosphotransferase system pathway showing differentially expressed genes upon cotreatment with loratadine and oxacillin compared to oxacillin only. The green to red legend represents log2 fold change in differential gene expression.

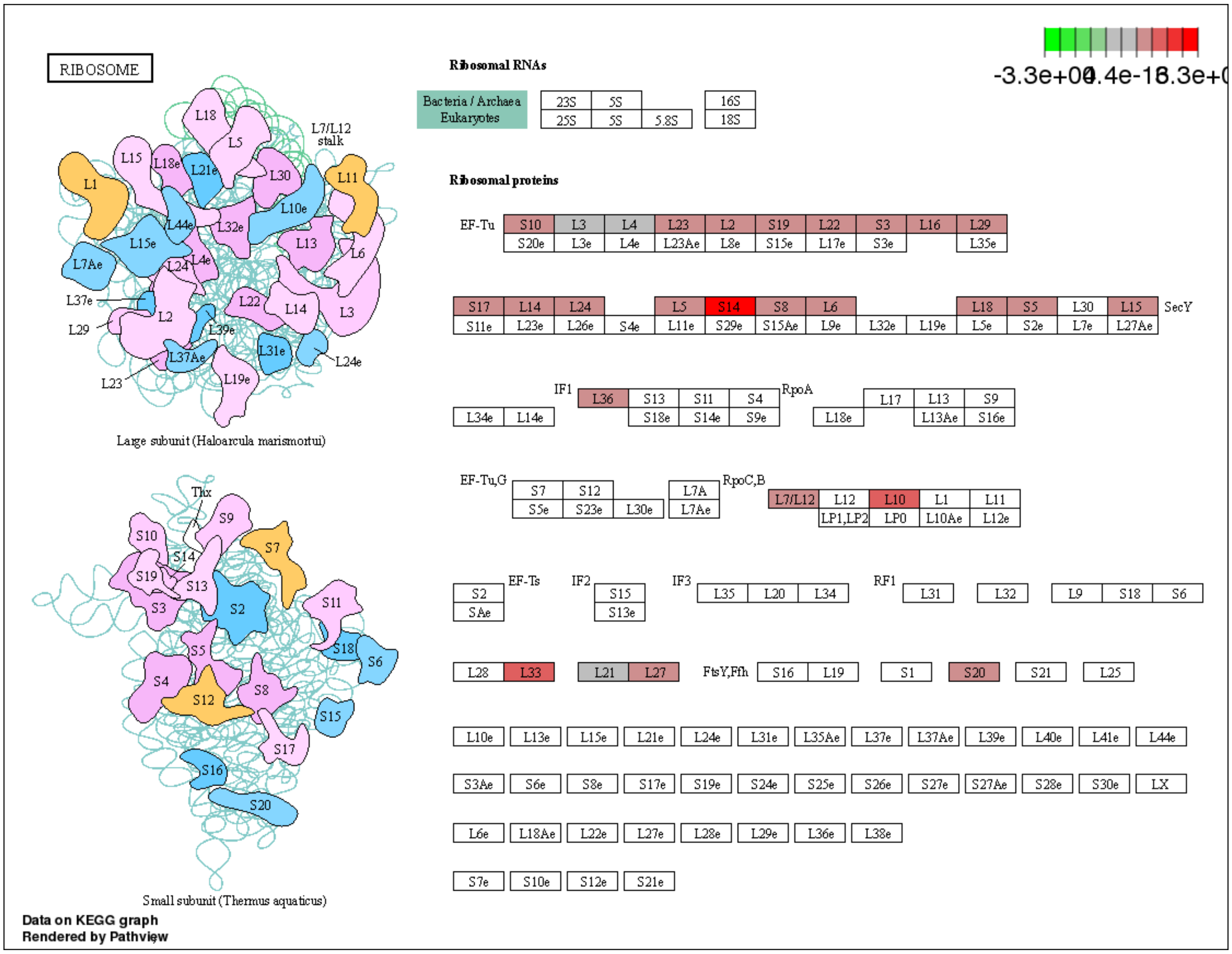

Supporting Figure S5: KEGG pathway map of the ribosomal pathway showing upregulated genes upon cotreatment of loratadine and oxacillin compared to oxacillin only. The green to red legend represents log2 fold change in differential gene expression.

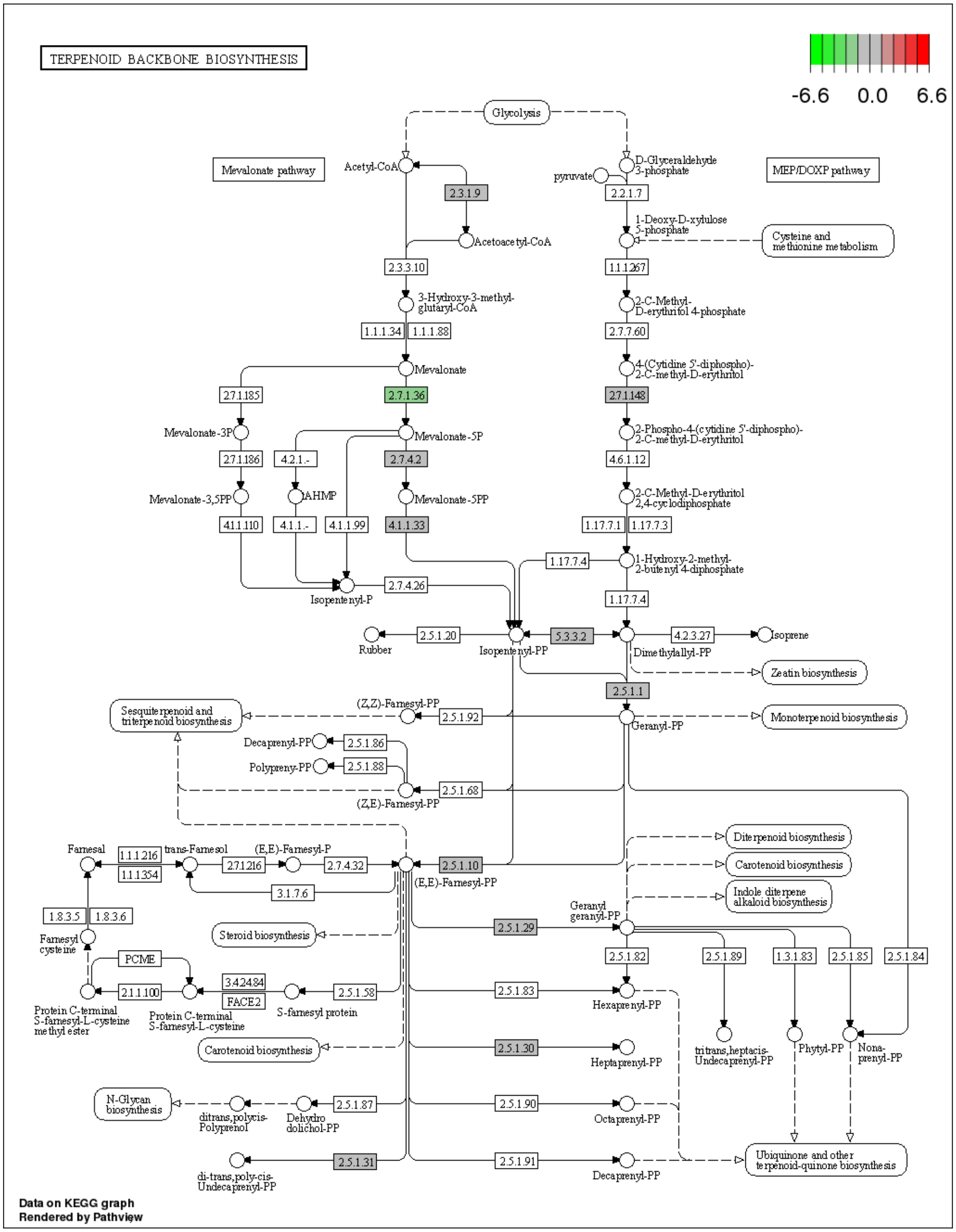

Supporting Figure S6: KEGG pathway map of the terpenoid backbone biosynthesis pathway showing downregulated genes upon cotreatment of loratadine and oxacillin compared to oxacillin only. The green to red legend represents log2 fold change in differential gene expression.

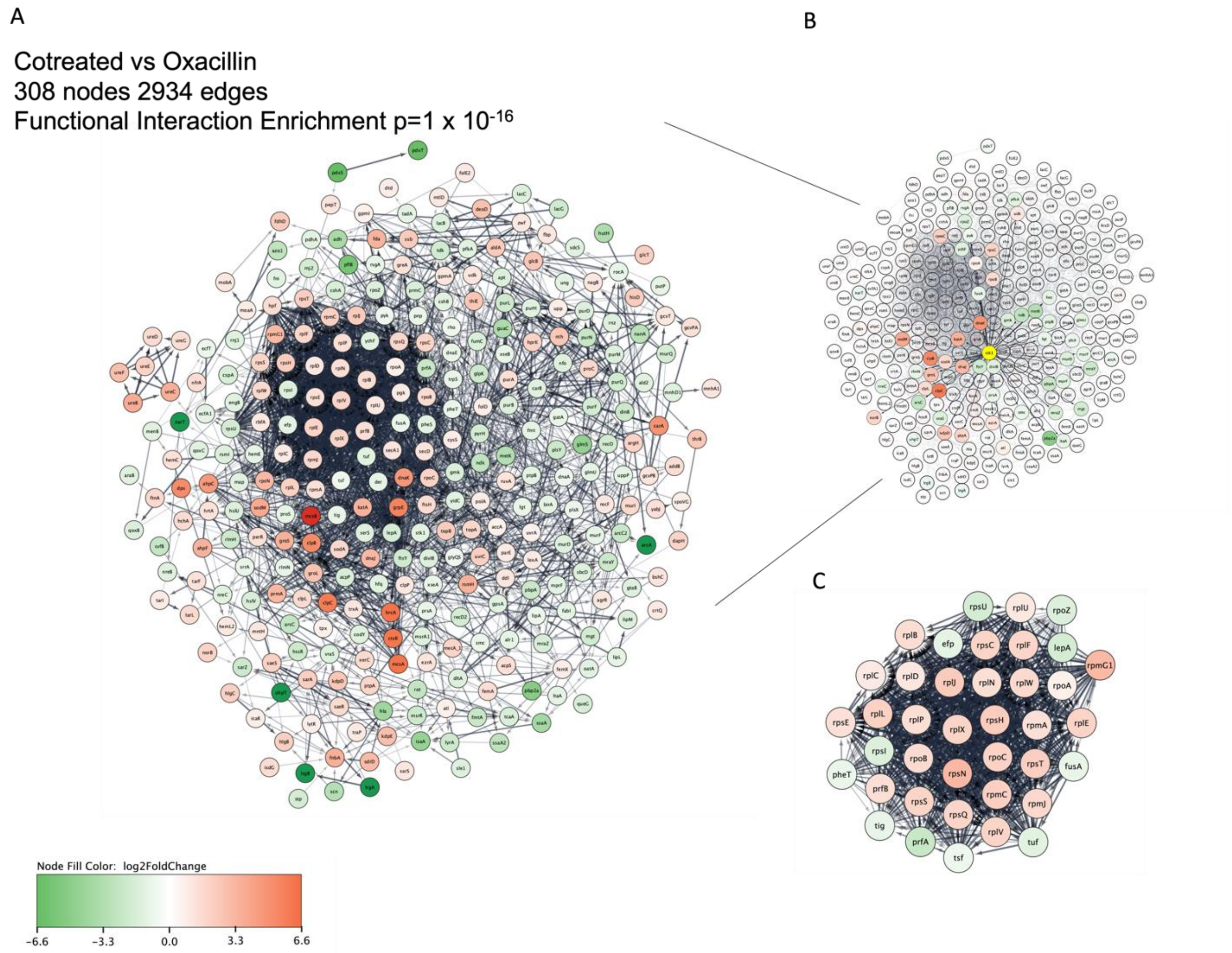

Supporting Figure S7: Protein interaction network of differentially expressed genes in samples cotreated with loratadine and oxacillin compared to oxacillin alone. (A) The legend green to red displays log2 fold change. (B) The network is identical, but has *stk1* highlighted in yellow, and only its first neighbors colored. (C) The top scoring MCODE cluster is shown. An interactive version of this network that can be viewed within Cytoscape is found on NDex at https://www.ndexbio.org/#/network/d63da07d-410c-11ee-aa50-005056ae23aa

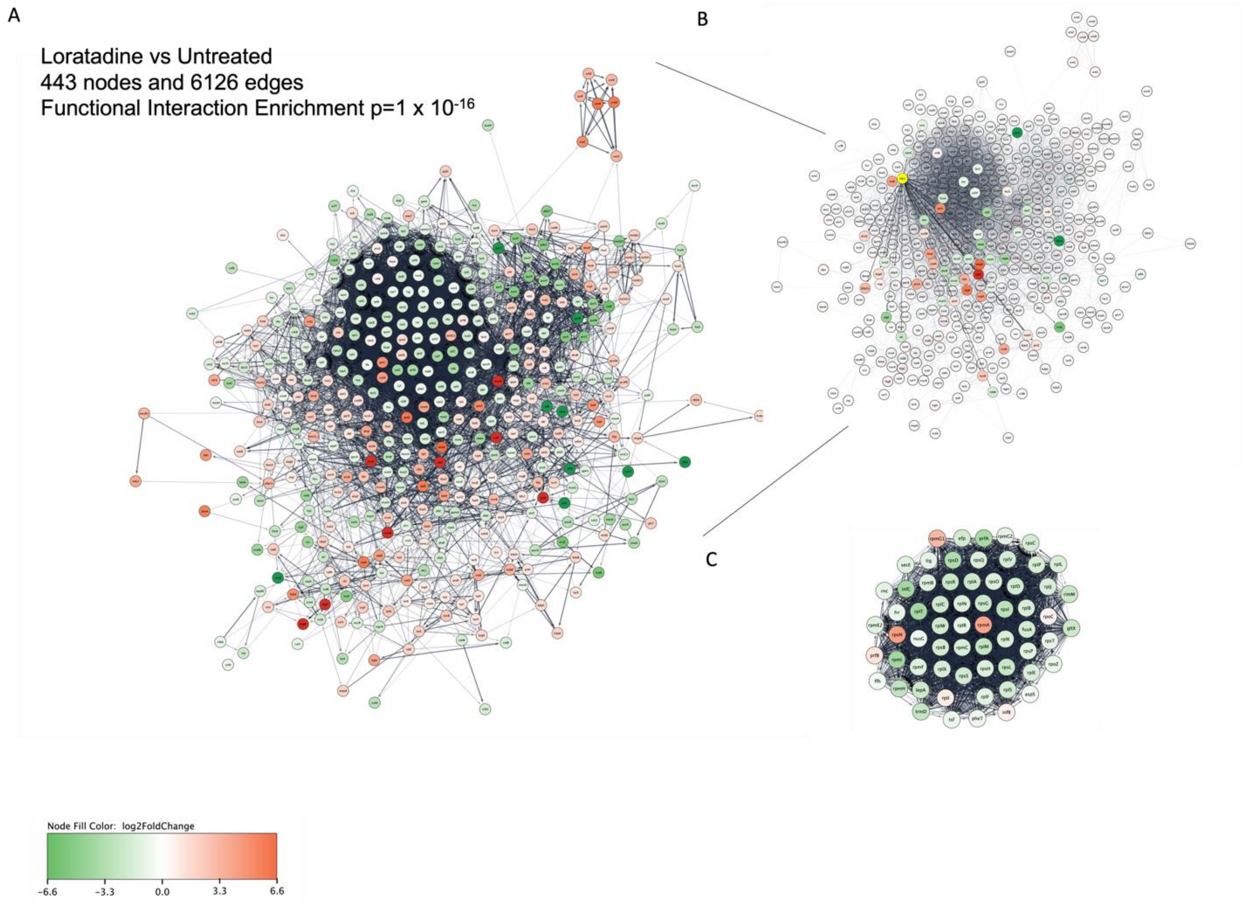

Supporting Figure S8: Protein interaction network of differentially expressed genes in samples treated with loratadine compared to untreated. (A)The legend green to red displays log2 fold change. (B) The network is identical, but has *stk1* highlighted in yellow, and only its first neighbors colored. (C) The top scoring MCODE cluster is shown. An interactive version of this network that can be viewed within Cytoscape is found on NDex at https://www.ndexbio.org/#/network/ee399c70-410c-11ee-aa50-005056ae23aa

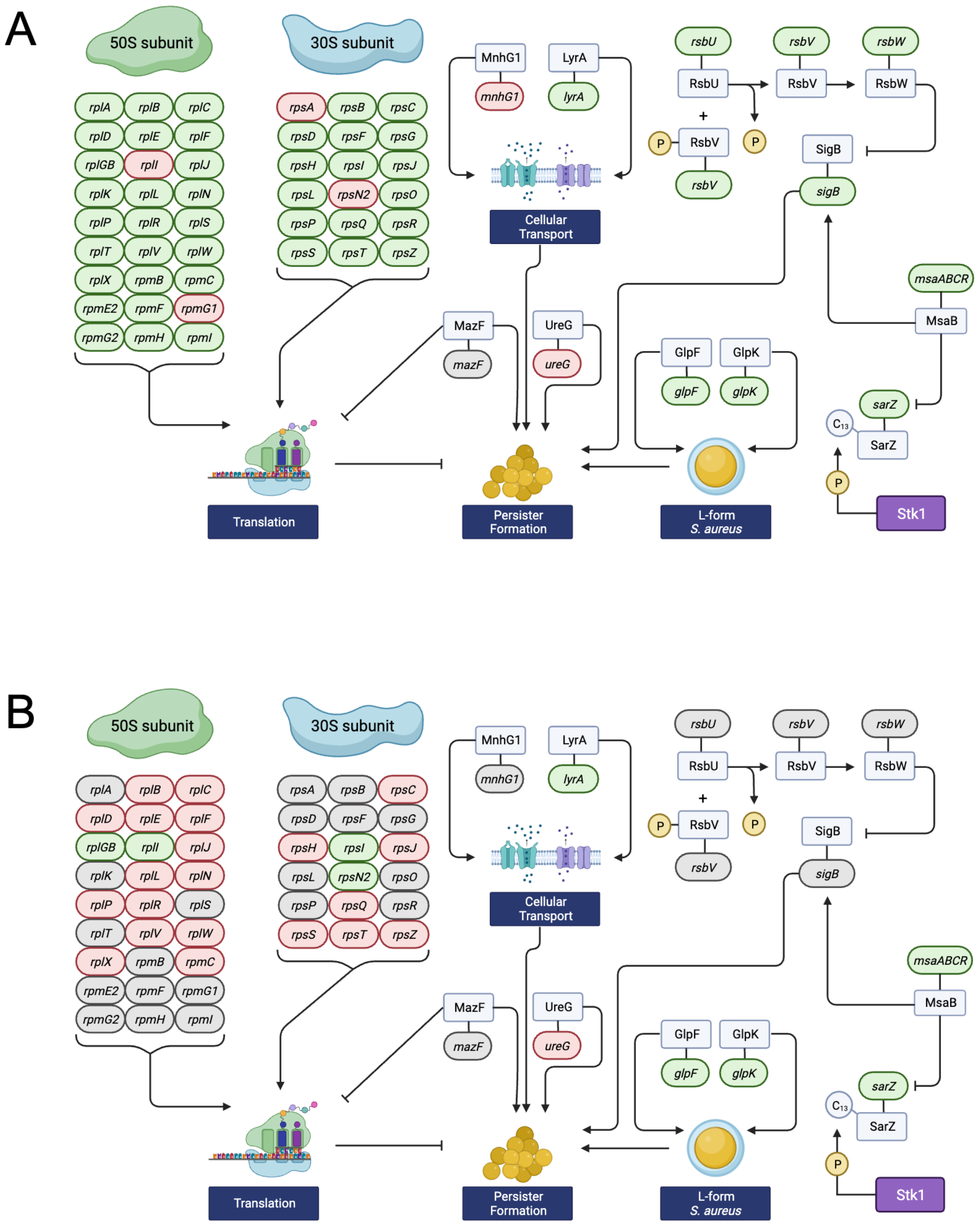

Supporting Information Figure S9. Loratadine treatment alone or in combination with oxacillin affects genes involved in persister formation. (A) In the top panel, comparisons were

made between gene expression in cells treated with loratadine alone as compared to untreated cells. (B) In the bottom panel, comparisons were made between gene expression in cells treated with a combination of loratadine and oxacillin as compared to oxacillin alone. Solid lines represent published and verified interactions. Dashed lines represent hypothesized interactions that have not been experimentally verified. Genes that displayed increased expression as compared to the control are shaded in red. Genes that displayed decreased expression as compared to the control are shaded in green. Many genes encoding proteins that make up both the large and small subunits of the bacterial ribosome are affected by treatment with loratadine alone or in combination with oxacillin. Alternate sigma factor B (SigB) is a major transcriptional regulator controlling the expression of virulence factors and genes associated with persister formation.[13] Expression of *sigB* is enhanced by MsaB, which also regulates expression of *sarZ*, whose protein SarZ is a known substrate of Stk1.[14, 15] At the protein level, SigB is inhibited by RsbW, which is activated by RsbV after dephosphorylation by RsbU.[16] Notably, loratadine treatment downregulated expression of *sarZ*, *msaABCR*, *rsbUVW*, and *sigB*. Treatment with loratadine alone and in combination with oxacillin lowered transcription of *glpF* and *glpK*. GlpF and GlpK have been identified as important proteins involved in glycerol uptake that are required for *S. aureus* to adopt the cell wall deficient L-form believed to be correlated to latent infections and persister formation.[4]

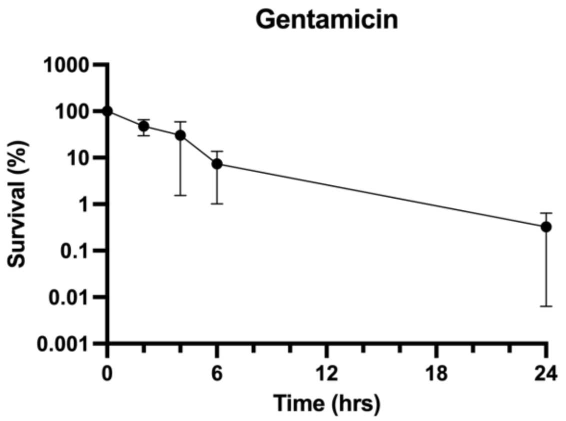

Supporting Figure S10: A biphasic kill curve with gentamicin treatment is observed. MRSA 43300 cells were treated with gentamicin at 10X MIC for 24 hours and aliquots were removed for enumeration on TSA spot plates. Results are shown as average % survival of the original time 0hr population from three biological replicates. Error bars represent the standard error of the mean.

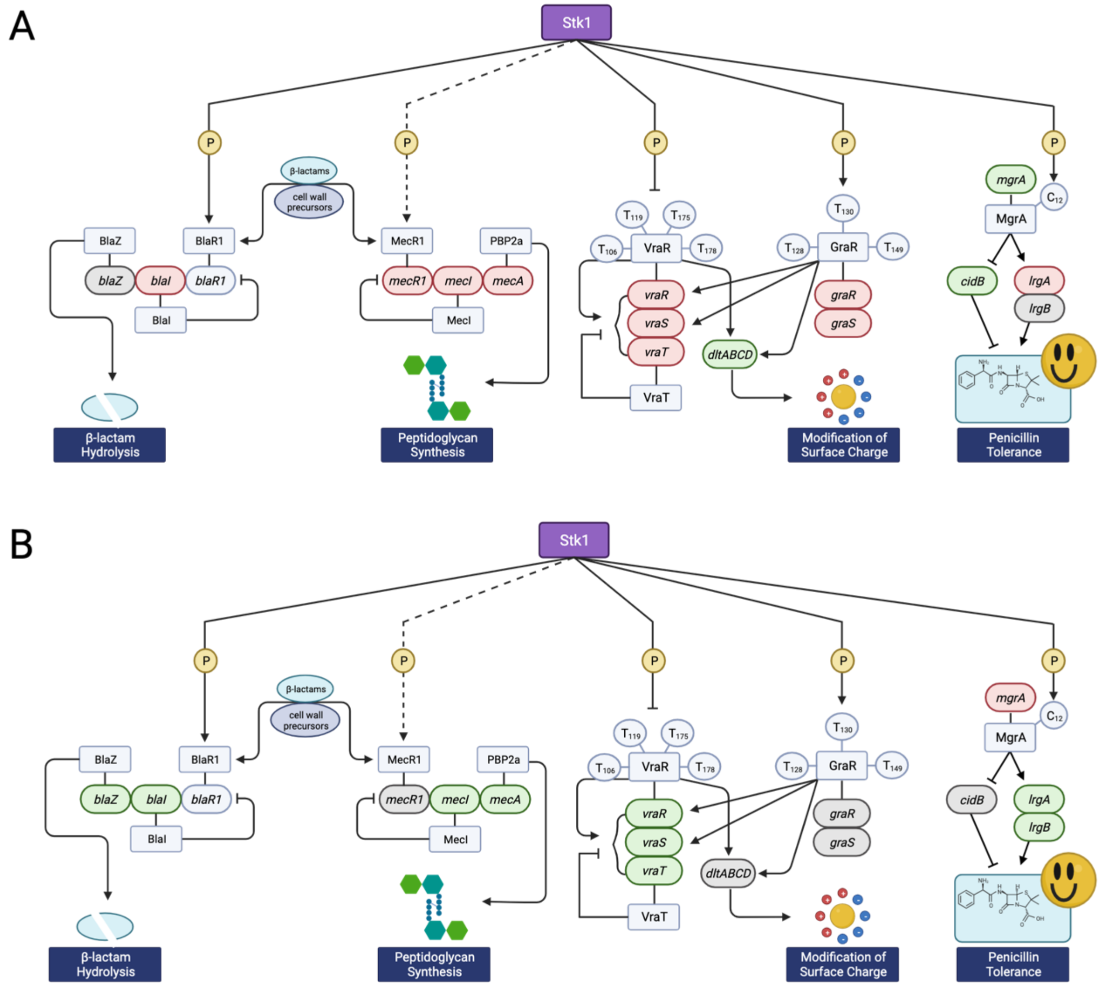

Supporting Information Figure S11. Loratadine treatment alone or in combination with oxacillin affects genes involved in antibiotic resistance and tolerance. (A) Comparisons were made between gene expression in cells treated with loratadine alone as compared to untreated cells. (B) Comparisons were made between gene expression in cells treated with a combination of loratadine and oxacillin as compared to oxacillin alone. Solid lines represent published and verified interactions. Dashed lines represent hypothesized interactions that have not been experimentally verified. Genes that displayed increased expression as compared to the control are shaded in red. Genes that displayed decreased expression as compared to the control are shaded in green. The genes in the *bla* operon encode proteins that sense and respond to β-lactam antibiotics by synthesizing β-lactamase. These genes are typically carried on transferable plasmids. Although they were not detected in our RNA-seq experiment, we have previously reported the effects of loratadine on these genes in ATCC 43300.[17] The *mec* operon encodes the genes that are canonically associated with methicillin resistance. In response to β-lactam treatment or an increased concentration of cell wall precursors, the *mec* operon synthesizes

PBP2a, a penicillin-binding protein with reduced affinity for β-lactam antibiotics, to rebuild and repair the cell wall.[17] The *vra* operon is associated with broad antibiotic resistance and tolerance activities. It encodes the two-component system VraRS, which regulates many genes involved in resistance to b-lactam antibiotics, vancomycin, daptomycin, and cationic peptides.[18-21] Similarly, GraRS has also been implicated in the regulation of antibiotic resistance genes and has also been shown to regulate expression of the *vraRST* operon to further regulate *S. aureus*'s response to antibiotic treatment.[22] Finally, *cidB* and *lrgAB* encode proteins that regulate murein hydrolase activity and whose expression correlates to reduced (*cidB*) or enhanced (*lrgAB*) tolerance to penicillin treatment.[23, 24]

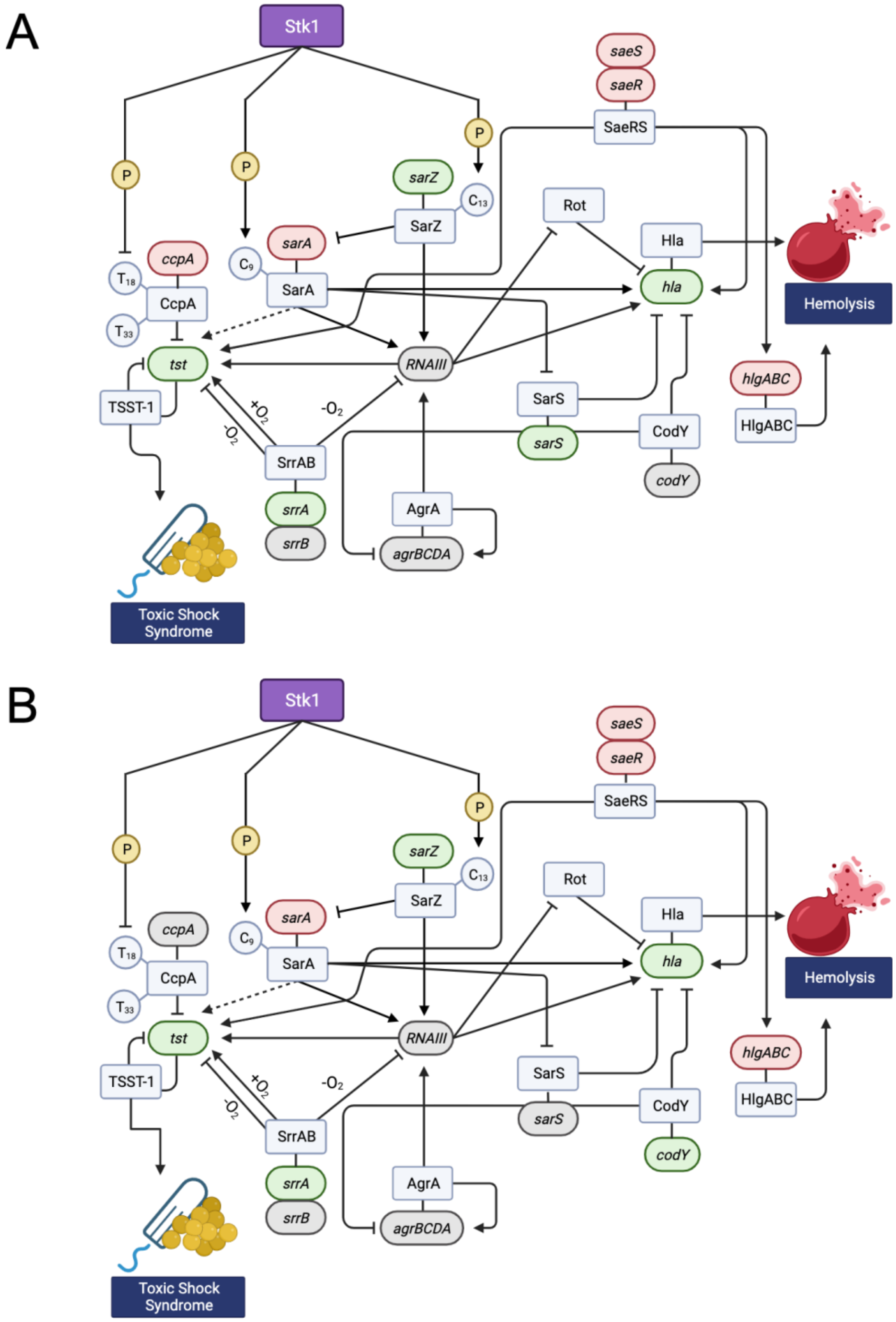

Supporting Information Figure S12. Loratadine treatment alone or in combination with oxacillin affects genes involved in virulence and toxin formation. (A) In the top panel, comparisons were made between gene expression in cells treated with loratadine alone as compared to untreated cells. (B) In the bottom panel, comparisons were made between gene expression in cells treated with a combination of loratadine and oxacillin as compared to

oxacillin alone. Solid lines represent published and verified interactions. Dashed lines represent hypothesized interactions that have not been experimentally verified. Genes that displayed increased expression as compared to the control are shaded in red. Genes that displayed decreased expression as compared to the control are shaded in green. CcpA is a glucose responsive transcriptional regulator that controls a number of genes linked to metabolism and virulence. Phosphorylation by Stk1 reduces CcpA's DNA binding affinity. In response to elevated glucose levels, CcpA represses expression of *tst*, the gene that encodes the toxic shock syndrome protein TSST-1.[25] SrrAB also regulates *tst* expression in response to oxygen levels. *RNAIII* and SaeRS both promote *tst* expression.[26] Expression of hemolysins, including HlgABC and Hla, are major contributors to *S. aureus* virulence and promote lysis of host cells.[27] Expression of *hla* is down-regulated by CodY, SarS, and Rot.[27] *RNAIII* is the major regulator of *hla* expression, with recent studies also implicating SarA and SaeR as up-regulators of expression.[27-29] SaeR also regulates *hlgABC* expression.[30]

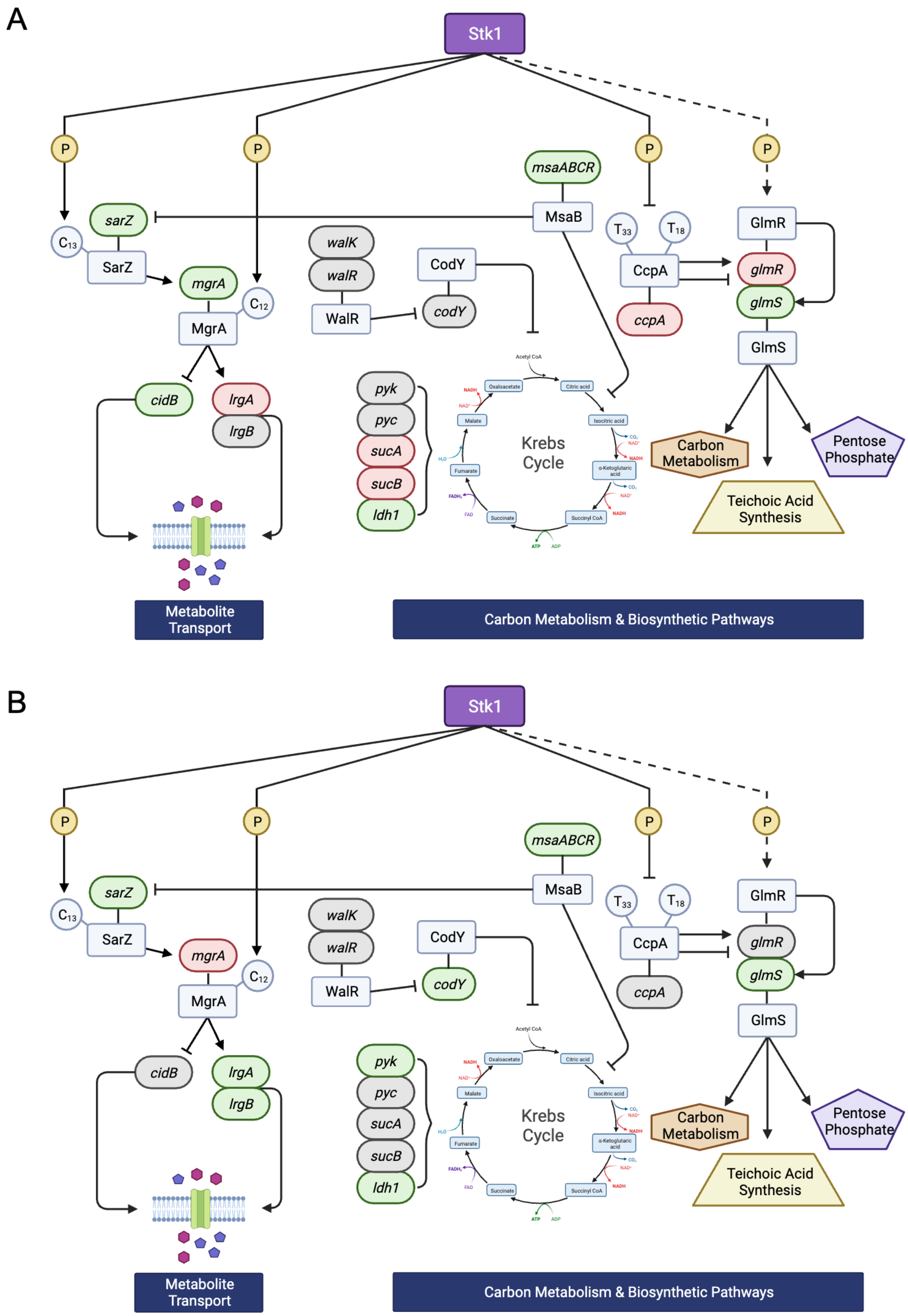
A
Stk1
P
P
P
P
msaABCR
MsaB
sarZ
C13
SarZ
mgrA
C12
MgrA
walK
walR
WalR
CodY
codY
T33
T18
CcpA
ccpA
GlmR
glmR
glmS
GlmS
cidB
lrgA
lrgB
pyk
pyc
sucA
sucB
ldh1
Krebs Cycle
Carbon Metabolism
Pentose Phosphate
Teichoic Acid Synthesis
Metabolite Transport
Carbon Metabolism & Biosynthetic Pathways
B
Stk1
P
P
P
P
msaABCR
MsaB
sarZ
C13
SarZ
mgrA
C12
MgrA
walK
walR
WalR
CodY
codY
T33
T18
CcpA
ccpA
GlmR
glmR
glmS
GlmS
cidB
lrgA
lrgB
pyk
pyc
sucA
sucB
ldh1
Krebs Cycle
Carbon Metabolism
Pentose Phosphate
Teichoic Acid Synthesis
Metabolite Transport
Carbon Metabolism & Biosynthetic Pathways

Supporting Information Figure S13. Loratadine treatment alone or in combination with oxacillin affects genes involved in metabolism. (A) In the top panel, comparisons were made between gene expression in cells treated with loratadine alone as compared to untreated cells. (B) In the bottom panel, comparisons were made between gene expression in cells treated with a combination of loratadine and oxacillin as compared to oxacillin alone. Solid lines represent published and verified interactions. Dashed lines represent hypothesized interactions that have not been experimentally verified. Genes that displayed increased expression as compared to the control are shaded in red. Genes that displayed decreased expression as compared to the control are shaded in green. CidB and LrgAB are known holin proteins that control transport of key metabolites in and out of the bacterial cell.[31] Expression of *cidB*, *lrgA*, and *lrgB* is regulated by MgrA, a known substrate of Stk1.[15, 32] Expression of *mgrA* is further regulated by SarZ, another verified substrate of Stk1.[33] CcpA is a glucose responsive transcriptional regulator that controls a number of genes linked to metabolism and virulence. Phosphorylation by Stk1 reduces CcpA's DNA binding affinity. CcpA differentially activates *glmR* expression in response to glucose levels, thereby regulating downstream metabolism pathways involved in carbon metabolism, cell wall biosynthesis, and the pentose phosphate pathway.[34] GlmR has also been proposed as a substrate of Stk1, though this has yet to be fully verified.[34] Several genes involved in the Krebs cycle were also affected by loratadine treatment alone and/or co-treatment with oxacillin. CodY, a known regulatory protein in *S. aureus*, exerts regulatory control on the Krebs cycle[35]; *codY* transcription was shown to be downregulated with co-treatment as compared to oxacillin alone.

A

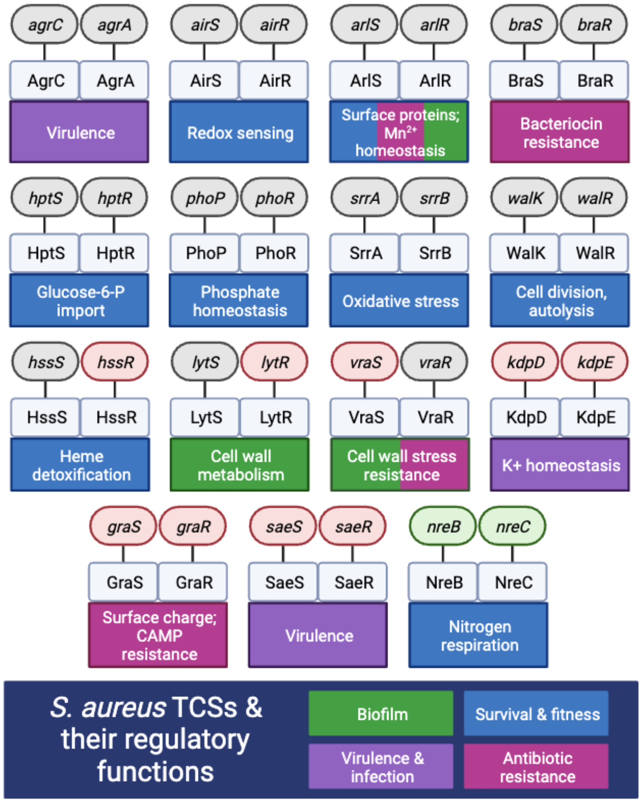
agrC
agrA
AgrC
AgrA
Virulence
airS
airR
AirS
AirR
Redox sensing
arlS
arlR
ArlS
ArlR
Surface proteins; Mn^{2+} homeostasis
braS
braR
BraS
BraR
Bacteriocin resistance
hptS
hptR
HptS
HptR
Glucose-6-P import
phoP
phoR
PhoP
PhoR
Phosphate homeostasis
srrA
srrB
SrrA
SrrB
Oxidative stress
walK
walR
WalK
WalR
Cell division, autolysis
hssS
hssR
HssS
HssR
Heme detoxification
lytS
lytR
LytS
LytR
Cell wall metabolism
vraS
vraR
VraS
VraR
Cell wall stress resistance
kdpD
kdpE
KdpD
KdpE
K+ homeostasis
graS
graR
GraS
GraR
Surface charge; CAMP resistance
saeS
saeR
SaeS
SaeR
Virulence
nreB
nreC
NreB
NreC
Nitrogen respiration
S. aureus TCSs & their regulatory functions
Biofilm
Survival & fitness
Virulence & infection
Antibiotic resistance

B

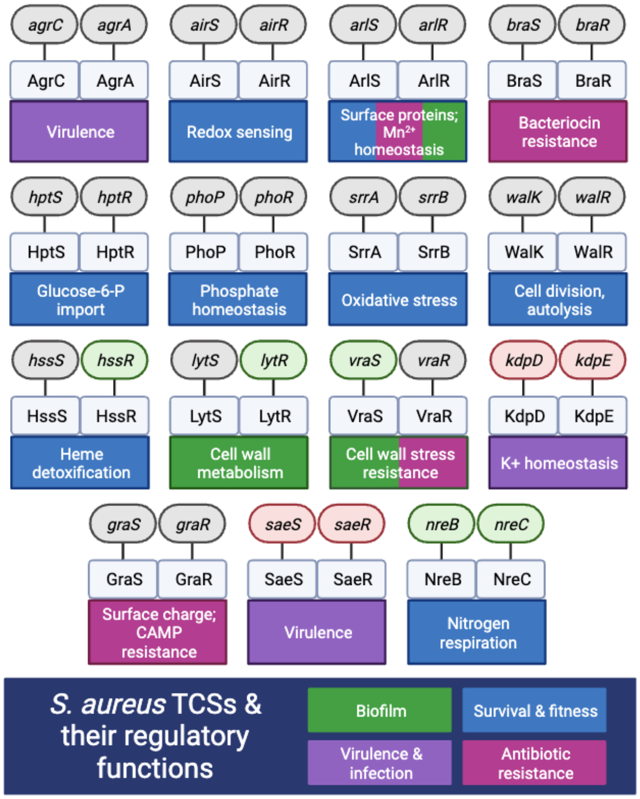
agrC
agrA
AgrC
AgrA
Virulence
airS
airR
AirS
AirR
Redox sensing
arlS
arlR
ArlS
ArlR
Surface proteins; Mn^{2+} homeostasis
braS
braR
BraS
BraR
Bacteriocin resistance
hptS
hptR
HptS
HptR
Glucose-6-P import
phoP
phoR
PhoP
PhoR
Phosphate homeostasis
srrA
srrB
SrrA
SrrB
Oxidative stress
walK
walR
WalK
WalR
Cell division, autolysis
hssS
hssR
HssS
HssR
Heme detoxification
lytS
lytR
LytS
LytR
Cell wall metabolism
vraS
vraR
VraS
VraR
Cell wall stress resistance
kdpD
kdpE
KdpD
KdpE
K+ homeostasis
graS
graR
GraS
GraR
Surface charge; CAMP resistance
saeS
saeR
SaeS
SaeR
Virulence
nreB
nreC
NreB
NreC
Nitrogen respiration
S. aureus TCSs & their regulatory functions
Biofilm
Survival & fitness
Virulence & infection
Antibiotic resistance

Supporting Information Figure S14. Loratadine treatment alone or in combination with oxacillin affects genes that encode for two component system proteins. (A) In the top panel, comparisons were made between gene expression in cells treated with loratadine alone as compared to untreated cells. (B) In the bottom panel, comparisons were made between gene expression in cells treated with a combination of loratadine and oxacillin as compared to oxacillin alone. Solid lines represent published and verified interactions. Dashed lines represent hypothesized interactions that have not been experimentally verified. Genes that displayed increased expression as compared to the control are shaded in red. Genes that displayed decreased expression as compared to the control are shaded in green. The general category of genes controlled by each TCS pair are indicated in the box below the protein names.[18, 36, 37] Regulatory functions related to survival and fitness are colored blue, functions related to biofilm are colored in green, functions related to virulence and infection are colored in purple, and functions related to antibiotic resistance are colored in burgundy. Where there are multiple regulatory functions, all applicable colors have been used.

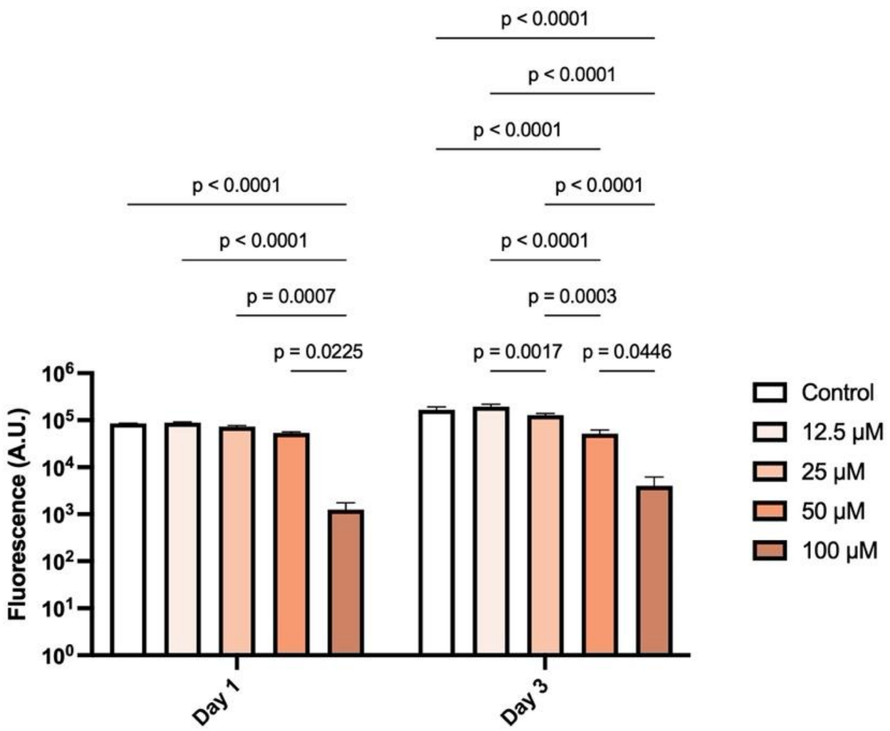

Supporting Figure S15: Loratadine is not toxic to human cells in culture at concentrations used in transcriptomic analysis. The y-axis shows fluorescence measured, which reports on cell viability due to redox of alamarBlue. The x-axis shows the loratadine concentration HEK 293 cells were exposed to.

A

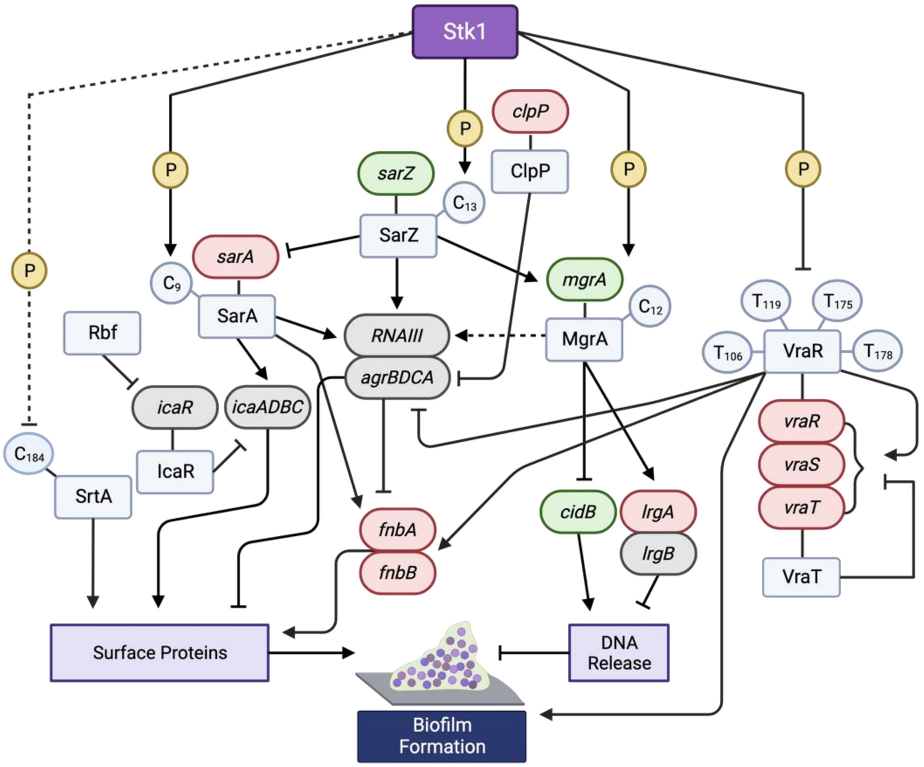
Stk1
P
P
clpP
ClpP
P
P
sarZ
C_{13}
SarZ
sarA
mgrA
C_{9}
C_{12}
SarA
RNAIII
MgrA
T_{119}
T_{175}
T_{106}
VraR
T_{178}
Rbf
agrBDCA
icaR
icaADBC
vraR
vraS
vraT
C_{184}
IcaR
SrtA
cidB
lrgA
lrgB
fnbA
fnbB
VraT
Surface Proteins
DNA Release
Biofilm Formation

B

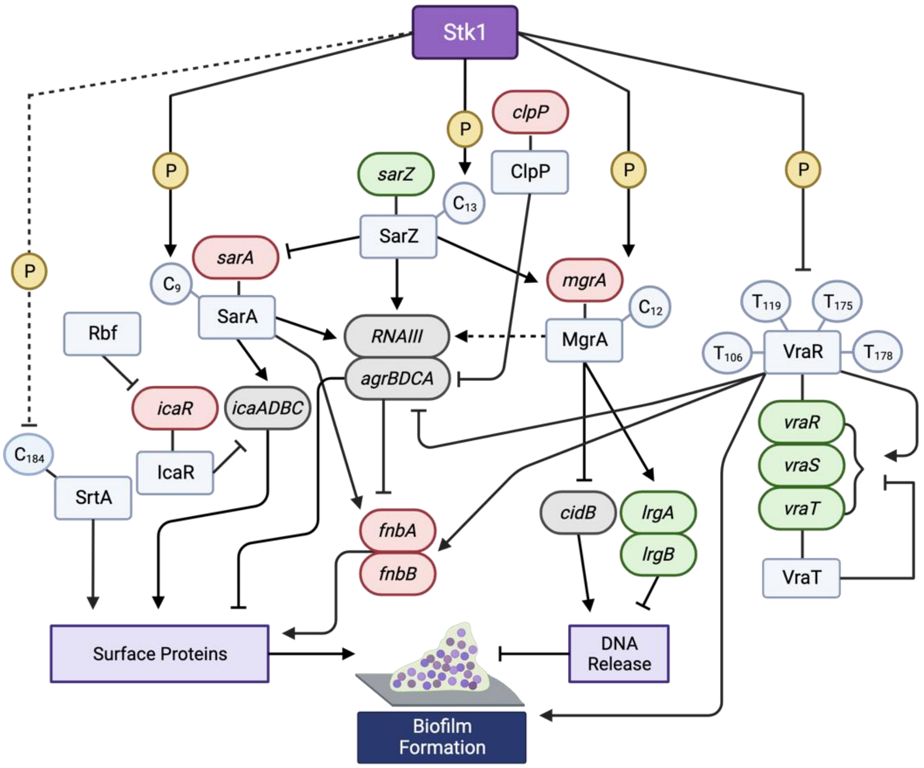
Stk1
P
P
clpP
ClpP
P
P
sarZ
C_{13}
SarZ
sarA
mgrA
C_{9}
C_{12}
SarA
RNAIII
MgrA
T_{119}
T_{175}
T_{106}
VraR
T_{178}
Rbf
agrBDCA
icaR
icaADBC
vraR
vraS
vraT
C_{184}
IcaR
SrtA
cidB
lrgA
lrgB
fnbA
fnbB
VraT
Surface Proteins
DNA Release
Biofilm Formation

Supporting Information Figure S16. Loratadine treatment alone or in combination with oxacillin affects genes involved in biofilm formation. (A) In the top panel, comparisons were made between gene expression in cells treated with loratadine alone as compared to untreated cells. (B) In the bottom panel, comparisons were made between gene expression in cells treated with a combination of loratadine and oxacillin as compared to oxacillin alone. Solid lines represent published and verified interactions. Dashed lines represent hypothesized interactions that have not been experimentally verified. Genes that displayed increased expression as compared to the control are shaded in red. Genes that displayed decreased expression as compared to the control are shaded in green. Biofilm formation is mediated by a complex interplay of genes that promote initial surface attachment and formation of extracellular matrix and genes that promote detachment and release from the matrix to promote formation of new biofilm colonies.[38-40] SarZ is a known substrate of Stk1 and is phosphorylated on Cys13.[15] Phosphorylation of SarZ leads to regulation of *mgrA*, *RNAIII*, *agr*, and *sarA* all of which are major regulators of genes involved in virulence and biofilm formation.[33] MgrA and SarA are substrates of Stk1 in their own right, and also regulate *agr* and *RNAIII*.[40, 41] SarA also regulates the *icaADBC* operon, which encodes surface proteins necessary for early adhesion and biofilm formation.[42] *icaADBC* is further regulated by IcaR, a transcriptional repressor that is differentially transcribed from the *icaADBC* operon.[43] MgrA inhibits transcription of *cidB*, which promotes DNA release and biofilm detachment, and promotes transcription of *lrgAB*, which inhibits DNA release.[23, 24, 42, 44] VraR, the response regulator of the VraRS TCS pair, inhibits *agr* and *RNAIII* and promotes biofilm formation. VraR also regulates its own transcription, which is repressed by VraT, encoded by the gene *vraT*, also known as *yvqF*.[45]

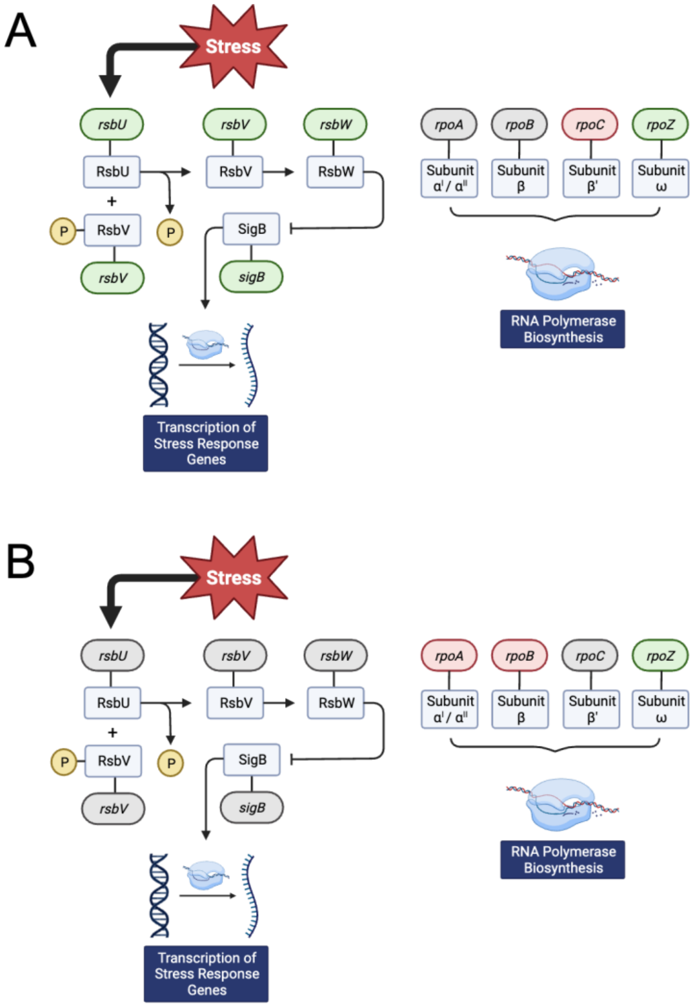

Supporting Information Figure S17. Loratadine treatment alone or in combination with oxacillin affects genes involved in global transcriptional regulation. (A) In the top panel, comparisons were made between gene expression in cells treated with loratadine alone as compared to untreated cells. (B) In the bottom panel, comparisons were made between gene expression in cells treated with a combination of loratadine and oxacillin as compared to oxacillin alone. Solid lines represent published and verified interactions. Dashed lines represent hypothesized interactions that have not been experimentally verified. Genes that displayed increased expression as compared to the control are shaded in red. Genes that displayed decreased expression as compared to the control are shaded in green. Alternate sigma factor B (SigB) is a major transcriptional regulator controlling the expression of virulence factors and genes associated with stress response.[13] At the protein level, SigB is inhibited by RsbW, which is activated by RsbV after dephosphorylation by RsbU.[16] Notably, loratadine treatment downregulated expression of *rsbUVW*, and *sigB*. Expression of the genes that encode proteins that form RNA polymerase were also significantly impacted by treatment with either loratadine alone or in combination with oxacillin.[46]

References

1. Viering, B.; Cunningham, T.; King, A.; Blackledge, M. S.; Miller, H. B., Brominated Carbazole with Antibiotic Adjuvant Activity Displays Pleiotropic Effects in MRSA's Transcriptome. *ACS Chemical Biology* **2022**.
2. Yarwood, J. M.; McCormick, J. K.; Paustian, M. L.; Kapur, V.; Schlievert, P. M., Repression of the Staphylococcus aureus accessory gene regulator in serum and in vivo. *J Bacteriol* **2002,** *184* (4), 1095-101.
3. Karimaei, S.; Kazem Aghamir, S. M.; Foroushani, A. R.; Pourmand, M. R., Antibiotic tolerance in biofilm persister cells of Staphylococcus aureus and expression of toxin-antitoxin system genes. *Microb Pathog* **2021,** *159*, 105126.
4. Han, J.; He, L.; Shi, W.; Xu, X.; Wang, S.; Zhang, S.; Zhang, Y., Glycerol uptake is important for L-form formation and persistence in Staphylococcus aureus. *PLoS One* **2014,** *9* (9), e108325.
5. Wang, W.; Chen, J.; Chen, G.; Du, X.; Cui, P.; Wu, J.; Zhao, J.; Wu, N.; Zhang, W.; Li, M.; Zhang, Y., Transposon Mutagenesis Identifies Novel Genes Associated with Staphylococcus aureus Persister Formation. *Front Microbiol* **2015,** *6*, 1437.
6. Peng, Q.; Guo, L.; Dong, Y.; Bao, T.; Wang, H.; Xu, T.; Zhang, Y.; Han, J., PurN Is Involved in Antibiotic Tolerance and Virulence in Staphylococcus aureus. *Antibiotics (Basel)* **2022,** *11* (12).
7. Yee, R.; Cui, P.; Shi, W.; Feng, J.; Zhang, Y., Genetic Screen Reveals the Role of Purine Metabolism in Staphylococcus aureus Persistence to Rifampicin. *Antibiotics (Basel)* **2015,** *4* (4), 627-42.
8. Li, L.; Li, Y.; Zhu, F.; Cheung, A. L.; Wang, G.; Bai, G.; Proctor, R. A.; Yeaman, M. R.; Bayer, A. S.; Xiong, Y. Q., New Mechanistic Insights into Purine Biosynthesis with Second Messenger c-di-AMP in Relation to Biofilm-Related Persistent Methicillin-Resistant Staphylococcus aureus Infections. *mBio* **2021,** *12* (6), e0208121.
9. Yee, R.; Cui, P.; Shi, W.; Feng, J.; Wang, J.; Zhang, Y., Identification of a novel gene argJ involved in arginine biosynthesis critical for persister formation in Staphylococcus aureus. *Discov Med* **2020,** *29* (156), 65-77.
10. Shang, Y.; Wang, X.; Chen, Z.; Lyu, Z.; Lin, Z.; Zheng, J.; Wu, Y.; Deng, Q.; Yu, Z.; Zhang, Y.; Qu, D., Staphylococcus aureus PhoU Homologs Regulate Persister Formation and Virulence. *Front Microbiol* **2020,** *11*, 865.
11. Xu, T.; Han, J.; Zhang, J.; Chen, J.; Wu, N.; Zhang, W.; Zhang, Y., Absence of Protoheme IX Farnesyltransferase CtaB Causes Virulence Attenuation but Enhances Pigment Production and Persister Survival in MRSA. *Front Microbiol* **2016,** *7*, 1625.
12. Sahukhal, G. S.; Pandey, S.; Elasri, M. O., msaABCR operon is involved in persister cell formation in Staphylococcus aureus. *BMC Microbiol* **2017,** *17* (1), 218.
13. Moisan, H.; Brouillette, E.; Jacob, C. L.; Langlois-Begin, P.; Michaud, S.; Malouin, F., Transcription of virulence factors in Staphylococcus aureus small-colony variants isolated from cystic fibrosis patients is influenced by SigB. *J Bacteriol* **2006,** *188* (1), 64-76.
14. Pandey, S.; Sahukhal, G. S.; Elasri, M. O., The msaABCR Operon Regulates the Response to Oxidative Stress in Staphylococcus aureus. *J Bacteriol* **2019,** *201* (21).
15. Sun, F.; Ding, Y.; Ji, Q.; Liang, Z.; Deng, X.; Wong, C. C.; Yi, C.; Zhang, L.; Xie, S.; Alvarez, S.; Hicks, L. M.; Luo, C.; Jiang, H.; Lan, L.; He, C., Protein cysteine phosphorylation of SarA/MgrA family transcriptional regulators mediates bacterial virulence and antibiotic resistance. *Proc Natl Acad Sci U S A* **2012,** *109* (38), 15461-6.
16. Pane-Farre, J.; Jonas, B.; Hardwick, S. W.; Gronau, K.; Lewis, R. J.; Hecker, M.; Engelmann, S., Role of RsbU in controlling SigB activity in Staphylococcus aureus following alkaline stress. *J Bacteriol* **2009,** *191* (8), 2561-73.
17. Cutrona, N.; Gillard, K.; Ulrich, R.; Seemann, M.; Miller, H. B.; Blackledge, M. S., From Antihistamine to Anti-infective: Loratadine Inhibition of Regulatory PASTA Kinases in Staphylococci

Reduces Biofilm Formation and Potentiates beta-Lactam Antibiotics and Vancomycin in Resistant Strains of Staphylococcus aureus. *ACS Infect Dis* **2019,** *5* (8), 1397-1410.
18. Worthington, R. J.; Blackledge, M. S.; Melander, C., Small-molecule inhibition of bacterial two-component systems to combat antibiotic resistance and virulence. *Future Med Chem* **2013,** *5* (11), 1265-84.
19. Dai, Y.; Chang, W.; Zhao, C.; Peng, J.; Xu, L.; Lu, H.; Zhou, S.; Ma, X., VraR Binding to the Promoter Region of agr Inhibits Its Function in Vancomycin-Intermediate Staphylococcus aureus (VISA) and Heterogeneous VISA. *Antimicrob Agents Chemother* **2017,** *61* (5).
20. Gao, C.; Dai, Y.; Chang, W.; Fang, C.; Wang, Z.; Ma, X., VraSR has an important role in immune evasion of Staphylococcus aureus with low level vancomycin resistance. *Microbes Infect* **2019,** *21* (8-9), 361-367.
21. Canova, M. J.; Baronian, G.; Brelle, S.; Cohen-Gonsaud, M.; Bischoff, M.; Molle, V., A novel mode of regulation of the Staphylococcus aureus Vancomycin-resistance-associated response regulator VraR mediated by Stk1 protein phosphorylation. *Biochem Biophys Res Commun* **2014,** *447* (1), 165-71.
22. Chen, L.; Wang, Z.; Xu, T.; Ge, H.; Zhou, F.; Zhu, X.; Li, X.; Qu, D.; Zheng, C.; Wu, Y.; Zhao, K., The Role of graRS in Regulating Virulence and Antimicrobial Resistance in Methicillin-Resistant Staphylococcus aureus. *Front Microbiol* **2021,** *12*, 727104.
23. Groicher, K. H.; Firek, B. A.; Fujimoto, D. F.; Bayles, K. W., The Staphylococcus aureus lrgAB operon modulates murein hydrolase activity and penicillin tolerance. *J Bacteriol* **2000,** *182* (7), 1794-801.
24. Rice, K. C.; Firek, B. A.; Nelson, J. B.; Yang, S. J.; Patton, T. G.; Bayles, K. W., The Staphylococcus aureus cidAB operon: evaluation of its role in regulation of murein hydrolase activity and penicillin tolerance. *J Bacteriol* **2003,** *185* (8), 2635-43.
25. Seidl, K.; Bischoff, M.; Berger-Bachi, B., CcpA mediates the catabolite repression of tst in Staphylococcus aureus. *Infect Immun* **2008,** *76* (11), 5093-9.
26. Andrey, D. O.; Jousselin, A.; Villanueva, M.; Renzoni, A.; Monod, A.; Barras, C.; Rodriguez, N.; Kelley, W. L., Impact of the Regulators SigB, Rot, SarA and sarS on the Toxic Shock Tst Promoter and TSST-1 Expression in Staphylococcus aureus. *PLoS One* **2015,** *10* (8), e0135579.
27. Tavares, A.; Nielsen, J. B.; Boye, K.; Rohde, S.; Paulo, A. C.; Westh, H.; Schonning, K.; de Lencastre, H.; Miragaia, M., Insights into alpha-hemolysin (Hla) evolution and expression among Staphylococcus aureus clones with hospital and community origin. *PLoS One* **2014,** *9* (7), e98634.
28. Gudeta, D. D.; Lei, M. G.; Lee, C. Y., Contribution of hla Regulation by SaeR to Staphylococcus aureus USA300 Pathogenesis. *Infect Immun* **2019,** *87* (9).
29. Xiong, Y. Q.; Willard, J.; Yeaman, M. R.; Cheung, A. L.; Bayer, A. S., Regulation of Staphylococcus aureus alpha-toxin gene (hla) expression by agr, sarA, and sae in vitro and in experimental infective endocarditis. *J Infect Dis* **2006,** *194* (9), 1267-75.
30. Liu, Q.; Yeo, W. S.; Bae, T., The SaeRS Two-Component System of Staphylococcus aureus. *Genes (Basel)* **2016,** *7* (10).
31. Endres, J. L.; Chaudhari, S. S.; Zhang, X.; Prahlad, J.; Wang, S. Q.; Foley, L. A.; Luca, S.; Bose, J. L.; Thomas, V. C.; Bayles, K. W., The Staphylococcus aureus CidA and LrgA proteins are functional holins involved in the transport of by-products of carbohydrate metabolism. *mBio* **2021,** *13* (1), e0282721.
32. Luong, T. T.; Dunman, P. M.; Murphy, E.; Projan, S. J.; Lee, C. Y., Transcription Profiling of the mgrA Regulon in Staphylococcus aureus. *J Bacteriol* **2006,** *188* (5), 1899-910.
33. Tamber, S.; Cheung, A. L., SarZ promotes the expression of virulence factors and represses biofilm formation by modulating SarA and agr in Staphylococcus aureus. *Infect Immun* **2009,** *77* (1), 419-28.
34. Liang, C.; Rios-Miguel, A. B.; Jarick, M.; Neurgaonkar, P.; Girard, M.; Francois, P.; Schrenzel, J.; Ibrahim, E. S.; Ohlsen, K.; Dandekar, T., Staphylococcusaureus transcriptome data and metabolic modelling investigate the interplay of ser/thr kinase PknB, Its phosphatase Stp, the glmR/yvcK regulon and the cdaA operon for metabolic adaptation. *Microorganisms* **2021,** *9* (10).

35. Majerczyk, C. D.; Dunman, P. M.; Luong, T. T.; Lee, C. Y.; Sadykov, M. R.; Somerville, G. A.; Bodi, K.; Sonenshein, A. L., Direct targets of CodY in Staphylococcus aureus. *J Bacteriol* **2010,** *192* (11), 2861-77.
36. Bleul, L.; Francois, P.; Wolz, C., Two-Component Systems of S. aureus: Signaling and Sensing Mechanisms. *Genes (Basel)* **2021,** *13* (1).
37. King, A.; Blackledge, M. S., Evaluation of small molecule kinase inhibitors as novel antimicrobial and antibiofilm agents. *Chem Biol Drug Des* **2021,** *98* (6), 1038-1064.
38. Blackledge, M. S.; Worthington, R. J.; Melander, C., Biologically inspired strategies for combating bacterial biofilms. *Curr Opin Pharmacol* **2013,** *13* (5), 699-706.
39. Schilcher, K.; Horswill, A. R., Staphylococcal Biofilm Development: Structure, Regulation, and Treatment Strategies. *Microbiol Mol Biol Rev* **2020,** *84* (3).
40. Patel, H.; Rawat, S., A genetic regulatory see-saw of biofilm and virulence in MRSA pathogenesis. *Front Microbiol* **2023,** *14*, 1204428.
41. Valle, J.; Toledo-Arana, A.; Berasain, C.; Ghigo, J. M.; Amorena, B.; Penades, J. R.; Lasa, I., SarA and not sigmaB is essential for biofilm development by Staphylococcus aureus. *Mol Microbiol* **2003,** *48* (4), 1075-87.
42. Cramton, S. E.; Gerke, C.; Schnell, N. F.; Nichols, W. W.; Gotz, F., The intercellular adhesion (ica) locus is present in Staphylococcus aureus and is required for biofilm formation. *Infect Immun* **1999,** *67* (10), 5427-33.
43. Cerca, N.; Brooks, J. L.; Jefferson, K. K., Regulation of the intercellular adhesin locus regulator (icaR) by SarA, sigmaB, and IcaR in Staphylococcus aureus. *J Bacteriol* **2008,** *190* (19), 6530-3.
44. Trotonda, M. P.; Tamber, S.; Memmi, G.; Cheung, A. L., MgrA represses biofilm formation in Staphylococcus aureus. *Infect Immun* **2008,** *76* (12), 5645-54.
45. Mlynek, K. D.; Callahan, M. T.; Shimkevitch, A. V.; Farmer, J. T.; Endres, J. L.; Marchand, M.; Bayles, K. W.; Horswill, A. R.; Kaplan, J. B., Effects of Low-Dose Amoxicillin on Staphylococcus aureus USA300 Biofilms. *Antimicrob Agents Chemother* **2016,** *60* (5), 2639-51.
46. Weiss, A.; Moore, B. D.; Tremblay, M. H. J.; Chaput, D.; Kremer, A.; Shaw, L. N., The omega Subunit Governs RNA Polymerase Stability and Transcriptional Specificity in Staphylococcus aureus. *J Bacteriol* **2017,** *199* (2).
